# Resisting the Final Line: Phenotypic Detection of Resistance to Last-Resort Antimicrobials in Gram-Negative Bacteria Isolated from Wild Birds in Northern Italy

**DOI:** 10.3390/ani15152289

**Published:** 2025-08-05

**Authors:** Maria Cristina Rapi, Joel Filipe, Laura Filippone Pavesi, Stefano Raimondi, Maria Filippa Addis, Maria Pia Franciosini, Guido Grilli

**Affiliations:** 1 Dipartimento di Medicina Veterinaria e Scienze Animali, Università degli Studi di Milano, Via dell’Università 6, 26900 Lodi, Italy; joel.soares@unimi.it (J.F.); laura.filippone@unimi.it (L.F.P.); filippa.addis@unimi.it (M.F.A.); guido.grilli@unimi.it (G.G.); 2 Centro Recupero Fauna Selvatica (CRAS)—“Bosco WWF di Vanzago”, Via delle 3 Campane, 20043 Vanzago, Italy; stefanoraimondi67@gmail.com; 3 Dipartimento di Medicina Veterinaria, Università degli studi di Perugia, Via San Costanzo 4, 06126 Perugia, Italy; mariapia.franciosini@unipg.it

**Keywords:** antimicrobial resistance, wild birds, last-line antimicrobials, HPCIAs, HCIAs, CIAs, ESBL, AmpC, One Health

## Abstract

Antimicrobial resistance (AMR) is a growing global health threat, with wild birds increasingly recognized as potential reservoirs of resistant pathogens and as sentinels of environmental AMR. This study investigated the occurrence and AMR profiles of Gram-negative bacteria isolated from wild birds that died at the Wildlife Rescue Center in Vanzago, Lombardy, in 2024. Cloacal swabs were collected from 112 birds representing various ecological categories. A total of 157 Gram-negative bacteria were isolated and identified, including clinically relevant genera and species, such as *Escherichia coli*, *Klebsiella pneumoniae*, *Enterobacter* spp., *Salmonella* spp., *Pseudomonas aeruginosa*, and *Acinetobacter baumannii*. Antimicrobial susceptibility testing revealed resistance to first-line and critically important antimicrobials, including those exclusively authorized for human use. Notably, a phenotype compatible with Extended-Spectrum Beta-Lactamase (ESBL) production was detected in four out of ten (40%) *K. pneumoniae* isolates. In addition, 20 out of the 157 (12.7%) isolated bacteria phenotypically exhibited a resistance profile indicative of AmpC beta-lactamase (AmpC) production, including *Enterobacter* spp. and *P. aeruginosa*. Resistance patterns were particularly interesting in birds with carnivorous, scavenging, or migratory-associated behaviors. These findings highlight the role of wild birds in the ecology and dissemination of antimicrobial-resistant bacteria (ARB) and highlight the need for wildlife-based AMR monitoring programs as part of a One Health approach.

## Simple Summary

Antimicrobial resistance represents a critical global health challenge, affecting human, animal, and environmental health. Wild birds, due to their mobility and ecological diversity, are increasingly recognized as potential carriers and spreaders of resistant bacteria. In this study, we examined wild birds that died at a wildlife rescue center in northern Italy to investigate the presence of Gram-negative bacteria in their intestinal tract and assess their antimicrobial resistance profiles. Cloacal samples were collected from 112 birds. A total of 157 Gram-negative bacterial isolates were identified, including clinically significant species. Notably, many isolates exhibited resistance to first-line and critically important antimicrobials, including those reserved for human medicine. Additionally, several isolates showed resistance patterns suggestive of mechanisms that further enhance their ability to withstand treatment. Resistance was more pronounced in carnivorous, scavenging, and migratory birds. These findings highlight the important role wild birds may play in the environmental dissemination of antimicrobial resistance and underscore the need for their inclusion in surveillance programs, supporting a One Health approach.

## 1. Introduction

Antimicrobial resistance (AMR) is widely recognized as one of the most urgent global health threats of the 21st century [1,2,3,4]. In 2019, AMR was responsible for an estimated 1.27 million deaths worldwide [5], and projections from the Review on Antimicrobial Resistance suggest that, by 2050, drug-resistant infections could cause up to 10 million deaths annually and result in a cumulative global economic loss of 100 trillion USD [6].

Although AMR is a natural occurring phenomenon, its rapid emergence and global spread have been accelerated by the misuse and overuse of antimicrobials in human medicine, veterinary practice, and agriculture, leading to the development and dissemination of resistance in both pathogenic and commensal bacteria [7,8,9,10]. To mitigate public health risks, the World Health Organization (WHO) introduced the list of Critically Important Antimicrobials (CIAs), updated in 2024 as the list of Medically Important Antimicrobials (MIAs), which categorizes drugs by their importance to human medicine [11,12]. These categories are intended to support risk-based decision-making by promoting the responsible use of antimicrobials in both human and veterinary medicine, ensuring that critically important drugs are not misused in food-producing animals while guiding the selection of veterinary agents with minimal AMR risk to humans [12]. In parallel, the Antimicrobial Advice ad hoc Expert Group (AMEG) categorizes antimicrobials into four groups (A–D) based on their suitability for veterinary use and public health risk [13]. This global framework reflects the principles of the One Health approach, which recognizes the close interconnection of human, animal, and environmental health [12,13,14]. Accordingly, AMR must be addressed not only in clinical settings, but also with consideration of environmental reservoirs, as antimicrobial-resistant bacteria (ARB) and antimicrobial-resistance genes (ARGs) have been widely detected in soil, surface waters, wastewater, and wild animals, including those not directly exposed to antimicrobial treatments [15,16]. In this context, wild birds are particularly important sentinels of AMR spread due to their high mobility, ecological diversity, and frequent interactions with both natural and anthropogenic environments, that make them effective bioindicators of environmental AMR contamination [15,17,18]. Several studies have reported the presence of bacteria resistant to last-resort antimicrobials in wild birds, raising concerns regarding human health and the dissemination of clinically relevant resistance mechanisms to diverse ecosystems [15,18,19]. For instance, *Escherichia coli* producing Extended-Spectrum Beta-Lactamases (ESBLs) and resistant to third-generation cephalosporins have been isolated from wild birds in both Europe and Asia [20,21]. Other studies have detected *Enterobacteriaceae* resistant to fluoroquinolones in aquatic birds, raptors, and passerines [15,18]. Carbapenem-resistant *E. coli* have also been identified in various wild bird species across multiple continents [15,22].

In this context, the present study aims to conduct a preliminary investigation, based solely on phenotypic detection methods, into the occurrence of AMR Gram-negative bacteria in cloacal samples collected from wild birds deceased at a wildlife rescue center in Lombardy region (northern Italy). These bacteria are known for their ability to acquire mobile genetic elements, such as plasmids, which makes them one of the most hazardous microorganisms for human health [23]. By considering antimicrobials critically important for human health, including those classified as “Authorized for use in humans only” and “Highest priority critically important antimicrobials” (HPCIAs) in the WHO MIAs list, as well as those assigned to the category A in the AMEG classification, this study contributes to the current knowledge on wildlife as potential reservoirs of AMR. Furthermore, it provides new insights into the phenotypically detectable presence of resistance to life-saving antimicrobials in wild birds in northern Italy, offering valuable data that may support One Health-based surveillance strategies, environmental risk assessments, and future policy development aimed at mitigating the spread of AMR.

## 2. Materials and Methods

### 2.1. Sample Collection

This study was conducted over a six-month period, from March to August 2024. All wild birds included either died spontaneously or were humanely euthanized at the wildlife rescue center WWF of Vanzago (Lombardy region), a facility dedicated to the sheltering and recovery of wild animals. The wildlife rescue center of Vanzago is the largest facility of its kind in northern Italy in terms of annual admissions and serves as a reference point for other regions in the country. It typically receives animals from over half of the provinces in Lombardy, covering nearly 70% of the region’s territory. At the Vanzago rescue center, every wild animal receives a medical evaluation upon admission. Each individual is assigned a unique identifier, and a dedicated clinical record is created. This record provides veterinarians and staff with detailed information on the individual, including clinical data, anamnesis, and any pharmacological treatments administered to the animal in relation to its clinical condition.

At this facility, euthanasia of wild birds is performed by the center’s veterinary medical director in accordance with national animal welfare legislation, and only in cases of severe illness, injury, or debilitation, or when release into the wild is not feasible. Therefore, no animals were deliberately or specifically sacrificed for the purposes of this study. Only individuals that had not received any pharmacological treatment were eligible for inclusion in the study.

### 2.2. Microbiological Analysis

Following death, the birds were transported and stored under refrigerated conditions to the Department of Veterinary Medicine and Animal Science of the University of Milan, for post mortem examination. During necropsy, intestinal content was collected for microbiological analysis. Each fecal sample underwent bacteriological examination for the detection of *Enterobacteriaceae*, intended here as the bacterial genera traditionally classified within this family prior to the reclassification (e.g., *Morganella* spp., *Proteus* spp., *Providencia* spp., *Hafnia* spp., and *Serratia* spp.) [24,25], and non-*Enterobacteriaceae*, defined in the present study as bacteria belonging to families and genera outside the order *Enterobacterales*. An enrichment phase was carried out by inoculating the fecal material into sterile tubes containing 10 mL of Buffered Peptone Water (BPW) (ThermoFisher, Oxoid, UK), followed by incubation at 37 °C for 24 h. Subsequently, the samples were streaked onto different media simultaneously, represented by Blood agar (ThermoFisher, Oxoid, UK), MacConkey agar (ThermoFisher, Oxoid, UK), and *Brilliance* UTI *Clarity* agar (ThermoFisher, Oxoid, UK), and incubated aerobically at 37 °C for 24 h. Following incubation, bacterial growth was assessed, and isolated colonies were identified at the species level using Matrix-Assisted Laser Desorption/Ionization–Time of Flight (MALDI-TOF) mass spectrometry with the MBT Microflex LT/SH MALDI-TOF mass spectrometer (Bruker Daltonik GmbH, Bremen, Germany), according to the protocol described by Rosa et al. [26].

For the isolation of *Salmonella* spp., samples were analyzed following the method outlined in the International Standards ISO 6579-1:2017 [27]. Colonies grown on differential media for *Salmonella* spp. detection were confirmed and identified only at the genus level using the MBT Microflex LT/SH MALDI-TOF mass spectrometer (Bruker Daltonik GmbH, Bremen, Germany). Serotyping of confirmed *Salmonella* spp. isolates was not performed.

### 2.3. Antimicrobial Susceptibility Testing (AST)

The disk diffusion method was performed as described in the literature [28,29], and according to the procedure described in the Clinical & Laboratory Standards Institute (CLSI) and provided by the European Committee on Antimicrobial Susceptibility Testing (EUCAST) [30,31]. One to four colonies were suspended in 5 mL of sterile distilled water to achieve a 0.5 McFarland turbidity standard. The suspension was then streaked onto Mueller–Hinton agar plates (ThermoFisher, Oxoid, UK), and isolates were tested against various antimicrobial agents using commercial antimicrobial disks (ThermoFisher, Oxoid, UK). The selection of antimicrobial panels was based on the study objectives and the bacterial orders identified, considering their intrinsic resistance (IR) profiles as defined by EUCAST and CLSI guidelines [30,32].

For *Enterobacterales* and *Burkholderiales*, susceptibility to 20 antimicrobial agents across 11 antimicrobial classes was evaluated: gentamicin (CN, 10 μg), cefotaxime (CTX, 5 μg), ceftazidime (CAZ, 10 μg), cefepime (FEP, 30 μg), cefoxitin (FOX, 30 μg), tetracycline (TE, 30 μg), doxycycline (DO, 30 μg), levofloxacin (LEV, 5 μg), ciprofloxacin (CIP, 5 μg), meropenem (MEM, 10 μg), imipenem (IPM, 10 μg), ampicillin (AMP, 10 μg), sulfamethoxazole/trimethoprim (SXT, 1.25/23.75 μg), amoxicillin/clavulanic acid (AMC, 20/10 μg), chloramphenicol (C, 30 μg), ceftazidime/avibactam (CZA, 10/4 μg), tigecycline (TGC, 15 μg), aztreonam (ATM, 30 μg), piperacillin (PRL, 30 μg) and piperacillin/tazobactam (TZP, 30/6 μg).

*Pseudomonadales* were tested with a modified panel, excluding FOX, AMP, AMC, and C, based on known IR profiles. Additionally, given the presence of breakpoints only on CLSI supplement M100 [30], disks containing 30 μg were used for all the tested third-generation cephalosporins (CTX, CAZ), as well as disks containing 100 μg and 100/10 μg for PRL and TZP, respectively, for *Acinetobacter* spp. AST. Similarly, in order to evaluate the antimicrobial resistance profile of *Pseudomonas aeruginosa*, the breakpoints established by the CLSI M100 supplement were preferred. Accordingly, disks containing 30 μg were used to test third-generation cephalosporins (CTX, CAZ), while disks containing 100 μg and 100/10 μg were employed for testing piperacillin (PRL) and piperacillin–tazobactam (TZP), respectively.

Bacterial susceptibility to each antimicrobial agent was first classified as susceptible (S), intermediate (I), and resistant (R) based on the breakpoints provided by the EUCAST and CLSI. Isolates showing intermediate susceptibility were then considered susceptible for interpretation purposes [33,34]. As a general rule, EUCAST breakpoints were preferably used [34,35]; if not available, CLSI supplement M100 breakpoints were used [30,36]. In cases lacking established breakpoints, epidemiological cut-off values (ECOFFs), literature data, or breakpoints for related species were used [37,38,39,40].

Additionally, all bacterial isolates were tested for colistin (CS, 0.016–256 μg/mL) susceptibility using MIC Test Strips (Liofilchem s.r.l., Roseto degli Abruzzi, Italy). Results were interpreted following manufacturer’s instructions [41]. The breakpoints used for the interpretation of phenotypic test results, along with their corresponding sources, are reported in the Supplementary Materials, Table S1.

Multi-drug resistant (MDR) bacteria were defined as those exhibiting resistance to at least one antimicrobial agent in three or more antimicrobial classes [42].

### 2.4. Phenotypic Detection of Resistance Profiles Compatible with ESBL and AmpC Beta-Lactamase Production

In order to identify resistance profiles consistent with ESBL production, all the isolates were streaked onto CHROMAgar™ ESBL plates (CHROMagar, Paris, France) according to the manufacturer’s instructions, and simultaneously were subjected to the double-disk synergy test (DDST). In accordance with EUCAST guidelines [43], the DDST was performed using ceftazidime (CAZ, 30 μg), cefotaxime (CTX, 30 μg), and amoxicillin/clavulanic acid (AMC, 30 μg) disks.

Resistance profiles suggestive of AmpC beta-lactamase production were phenotypically assessed using the cefoxitin screening test, followed by confirmation via the disk approximation test [44,45]. As described in the literature, AmpC-compatible (cAmpC) profile detection was performed by inoculating Mueller–Hinton agar plates (ThermoFisher, Oxoid, UK) with the tested isolate. Bacterial isolates with a cefoxitin zone diameter of less than 18 mm in the disk diffusion method were considered potential AmpC producers and were further examined using phenotypic testing via the disk approximation test, employing ceftazidime (CAZ, 30 μg), imipenem (IPM, 10 μg), cefoxitin (FOX, 30 μg), and amoxicillin/clavulanic acid (AMC, 20/10 μg) disks. Isolates for which the results of these tests were ambiguous or difficult to interpret were further analyzed using MASTDISCS^®^ *Combi* AmpC Detection Set (Mast Group Ltd., UK); results were interpreted following manufacturer’s instructions.

### 2.5. Quality Control

To ensure the reliability of incubation conditions and culture media performance, as well as the quality and accuracy of AST procedures, American Type Culture Collection (ATCC) standard reference (*E. coli* ATCC 25922) and National Collection of Type Cultures (NCTC) (*E. coli* NCTC 13846) strains were used.

### 2.6. Sample Size Assesment

Sample size was calculated using the formula described by Thrusfield [46]. In the absence of prior regional data, a conservative estimate of the lowest reported prevalence of resistance to key antimicrobials of critical importance in human medicine, particularly colistin, was used. Based on a review of the available literature, and focusing on this molecule, the lowest reported prevalence of resistance in wild bird-associated bacteria was 0.85% [15,47,48,49,50,51,52]. A 95% confidence level (CI) and ±2% precision were chosen to ensure robust detection of rare resistance profiles. This yielded a required minimum sample size of 81 birds to ensure sufficient power for detecting low-frequency resistance patterns.

### 2.7. Statistical Analysis

All statistical analyses were conducted in SPSS version 29.0 (IBM Corp., Armonk, NY USA). Initially, each potential predictor, age class (juvenile vs. adult), feeding behavior (granivorous, omnivorous, carnivorous, etc.), spatial behavior (sedentary vs. migratory), and habitat (synanthropic vs. wildland species), was tested in a univariable logistic regression against each binary outcome (major pathogen isolation, *Salmonella* presence, or AMR pattern in bacteria isolated from different wild birds’ categories), and those variables achieving a *p*-value below 0.20 were retained for multivariable modeling. In the subsequent multivariable logistic regression, we assessed and excluded any predictors with variance inflation factors ≥3 to guard against multicollinearity, and we confirmed that each retained variable contributed significantly to model deviance using likelihood-ratio tests. Final models reported odds ratios (ORs), with statistical significance set at *p* ≤ 0.05 and *p*-values between 0.05 and 0.10 noted as trends toward significance. Model calibration was evaluated by the Hosmer–Lemeshow goodness-of-fit test, where a non-significant result (*p* > 0.05) indicated acceptable fit, and any case with missing data on included variables was excluded listwise.

## 3. Results

Over the six-month sampling period, 112 wild birds representing 37 different species belonging to 13 orders were collected (Table 1).

### 3.1. Bacterial Isolation and Identification

From a total of 112 wild bird specimens sampled, 254 bacteria were isolated and identified. Of these, 157 (61.8%) were Gram-negative bacteria belonging to three taxonomic orders, namely *Enterobacterales* (*n* = 148/157, 94.3%), *Pseudomonadales* (*n* = 7/157, 4.4%), and *Burkholderiales* (*n* = 2/157, 1.3%) (Supplementary Materials, Figure S1).

Among *Enterobacterales*, the most frequently isolated species was *Escherichia coli* (*n* = 66/148, 44.6%), followed by *Proteus mirabilis* (*n* = 31/148, 20.9%), *Salmonella* spp. (*n* = 11/148, 7.4%), and *Klebsiella pneumoniae* (*n* = 10/148, 6.7%). Other isolated potentially pathogenic species within this order included *Enterobacter hormaechei* (*n* = 5), *Klebsiella oxytoca* (*n* = 3), and *Serratia marcescens* (*n* = 1), as reported in Supplementary Materials, Figure S1. Within the *Pseudomonadales* order, *Pseudomonas aeruginosa* accounted for the majority of isolates (*n* = 5/7, 71.4%). Additional clinically relevant species within this order included *Acinetobacter pittii* (*n* = 1; 14.3%) and *Acinetobacter baumannii* (*n* = 1; 14.3%) (Supplementary Materials, Figure S1). With reference to the *Burkholderiales* order, only one isolate each of *Comamonas kerstersii* and *Achromobacter mucicolens* was recovered from the sampled animals, as shown in Supplementary Materials, Figure S1.

**Figure 1:**
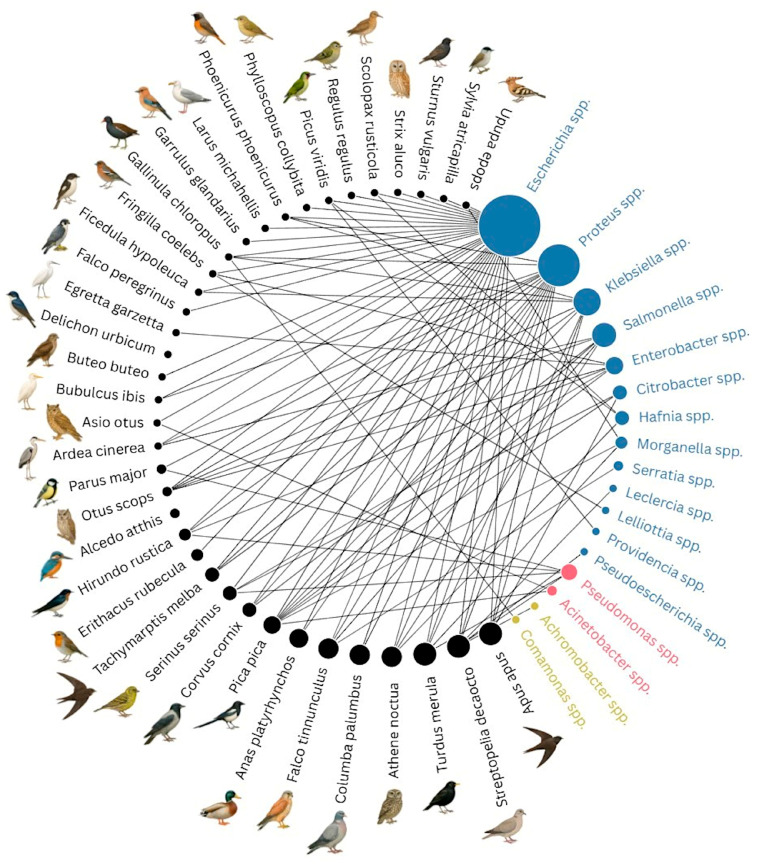
Network graph showing associations between wild bird species and bacterial genera isolated from cloacal samples. The circular representation displays connections between wild bird species (black nodes with stylized illustrations on the left) and the bacterial genera isolated (colored nodes on the right). The size of the bacterial nodes is proportional to the total number of isolates per genus. Similarly, the size of black nodes is proportional to the number of sampled birds belonging to the indicated species. Node color represents bacterial taxonomic order: *Enterobacterales* (dark blue), *Pseudomonadales* (pink), and *Burkholderiales* (yellow). Grey lines indicate the presence of at least one bacterial isolate of the corresponding genus isolated from the linked bird species. For some species, no Gram-negative bacteria were isolated from fecal samples.

Among the most frequently isolated bacterial genera, *Escherichia* spp. was identified in a wide variety of hosts (18 out of 37 wild bird species sampled), making it the genus with the highest number of associations with different bird species. Other genera isolated from several bird species included *Proteus* spp. (11 wild bird species), *Klebsiella* spp., and *Salmonella* spp. (each from 8 different avian species). These findings are reported in Supplementary File, Table S2, and graphically represented in Figure 1.

### 3.2. Phenotypic AMR Profile of Bacteria Isolates

The isolates showed a variety of antimicrobial resistance patterns, as reported in Supplementary Materials, Tables S3–S5.

**Figure 2:**
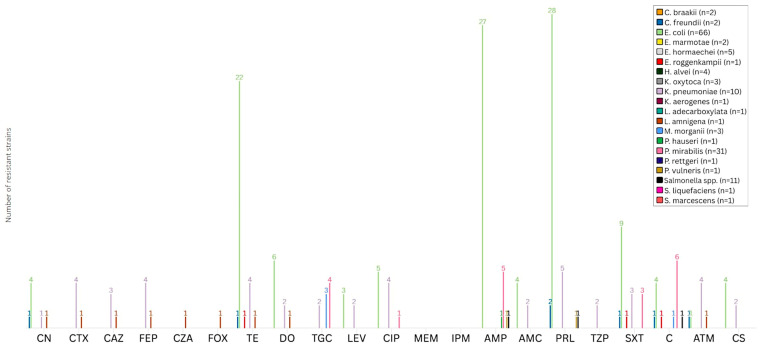
Acquired antimicrobial resistance profiles among *Enterobacterales* isolates. Bar chart illustrating the number of *Enterobacterales* isolates exhibiting acquired resistance to selected antimicrobial agents. Intrinsic resistance traits were excluded from the analysis. Antimicrobial molecules are reported along the *x*-axis, while the *y*-axis indicates the number of resistant isolates. Bar colors correspond to individual bacterial species, as detailed in the legend on the right. CN: gentamicin; CTX: cefotaxime; CAZ: ceftazidime; FEP: cefepime; CZA: ceftazidime/avibactam; FOX: cefoxitin; TE: tetracycline; DO: doxycycline; TGC: tigecycline; LEV: levofloxacin; CIP: ciprofloxacin; MEM: meropenem; IPM: imipenem; AMP: ampicillin; AMC: amoxicillin/clavulanic acid; PRL: piperacillin; TZP: piperacillin/tazobactam; SXT: sulfamethoxazole/trimethoprim; C: chloramphenicol; ATM: aztreonam; CS: colistin.

Regarding *Enterobacterales*, a significant range of resistance profiles among different antimicrobial types and bacterial species was identified, as illustrated in Figure 2 and detailed in Supplementary Materials, Table S6. The total number of isolates resistant to the tested antimicrobial agents was determined excluding those whose resistance is intrinsically determined. Resistance to CN was observed in only 4.7% (*n* = 7/148) of isolates, while third- and fourth-generation cephalosporins displayed resistance rates ranging from 2.7% (*n* = 4/148) for CAZ to 3.4% (*n* = 5/148) for both CTX and FEP, with most of the resistant isolates belonging to *K. pneumoniae*. Notably, resistance to CZA was low (0.7%), with only one isolate of *L. amnigena* classified as not susceptible. Moreover, resistance to fluoroquinolones was moderate, with 6.7% (*n* = 10/148) and 3.4% (*n* = 5/148) of isolates resistant to CIP and LEV, respectively, mainly involving *E. coli* and *K. pneumoniae*. In contrast, no resistance to carbapenem was detected. Regarding penicillins, AMP and PRL displayed higher resistance levels, with 23.6% (*n* = 35/148) and 25% (*n* = 37/148) of all the isolates classified as resistant. On the opposite end, resistance to TZP and AMC remained limited, at 1.3% (*n* = 2/148) and 4% (*n* = 6/148), respectively. Among tetracyclines, DO showed lower resistance rates (6.1%, *n* = 9/148) compared to TE (19.6%, *n* = 29/148), with 33% (*n* = 22/66) of *E. coli* and 40% (*n* = 4/10) of *K. pneumoniae* isolates being resistant to the latter molecule. Resistance to TGC was observed in 6.1% (*n* = 9/148) of the *Enterobacterales* isolates. Resistance to SXT and C was moderate, recorded at 11.5% (*n* = 17/148) and 9.5% (*n* = 14/148). Regarding monobactam, resistance to ATM was identified in 4.7% of isolates (*n* = 7/148), primarily in *K. pneumoniae*. Resistance to CS was detected in 6 out of 148 (4%) tested *Enterobacterales*, with 6% of *E. coli* (*n* = 4/66) and 20% of *K. pneumoniae* (*n* = 2/10) isolates being resistant to this molecule.

Antimicrobial susceptibility profiles of *Pseudomonadales* are summarized in Table S7 in Supplementary Materials. Resistance among this group was overall low, with no acquired resistance detected among the *P. aeruginosa* isolates. For *A. baumannii* and *A. pittii*, resistance was limited, with both isolates resistant to CTX, which resulted in a total CTX resistance rate of 28.6% (*n* = 2/7) within the order. Additionally, *A. pittii* was reported as resistant to PRL.

AST results of the two isolates within the *Burkholderiales* order revealed no acquired resistance to the tested antimicrobial agents (Supplementary Materials, Table S8). However, interpretation of the results was limited by the lack of defined breakpoints for most agents; in particular, for *C. kerstersii*, no interpretive analysis could be conducted.

Of the 157 Gram-negative bacteria tested, 24 (15.3%) exhibited a MDR profile. All MDR isolates belonged to the order *Enterobacterales* (Supplementary Materials, Table S3); among these, 50% (*n* = 12/24) were *E. coli* isolates, 20% (*n* = 5/24) were *K. pneumoniae* isolates, and 12% (*n* = 3/24) were *P. mirabilis* isolates.

### 3.3. Phenotypic Detection of Resistance Profiles Compatible with ESBL and AmpC Beta-Lactamase Production

Of the 157 isolated Gram-negative bacteria, 3.2% (*n* = 5/157) showed a resistance profile compatible with ESBL production (cESBL) and 12.7% (*n* = 20/157) presented a cAmpC profile.

A total of four cESBL isolates were *Enterobacterales* (*n* = 4/148, 3.4%), all of them belonging to the *K. pneumoniae* species. These exhibited resistance to third- and fourth-generation cephalosporins, penicillins, and aztreonam (Supplementary Materials, Table S3). The remaining cESBL was identified as *A. pittii*, belonging to the order *Pseudomonadales* (Supplementary Materials, Table S4). Concerning cAmpC, five isolates belonged to the order *Pseudomonadales*, specifically *P. aeruginosa* (Supplementary Materials, Table S4), with the remaining cAmpC isolates belonged to the *Enterobacterales* order (Supplementary Materials, Table S3).

A substantial proportion of the cESBL and cAmpC isolates were also classified as MDR, as detailed in Supplementary Materials, Tables S3–S5.

### 3.4. Factors Associated with Pathogen Isolation and Antimicrobial Resistance

Binary logistic regression analysis showed that age was associated with a borderline reduced risk of major pathogen isolation, with adults being less likely than juveniles to carry pathogens (OR = 0.55, *p* = 0.058). Living in wild habitats also showed a trend toward protection (OR = 0.45, *p* = 0.10) (Supplementary Materials, Table S9). Regarding *Salmonella* isolation, granivorous diets were significantly protective compared to carnivorous/scavenger diets (OR = 0.14, *p* = 0.004), while non-wild (anthropized) habitats showed a strong trend toward increased risk (OR = 12.29, *p* = 0.063), as reported in Supplementary Materials, Table S10. In the analysis of antimicrobial resistance in *E. coli*, only spatial behavior was significantly associated with piperacillin resistance (OR = 0.28, *p* = 0.048), indicating that sedentary individuals were less likely to carry resistant strains (Supplementary Materials, Table S11). No other significant associations were found for other specific bacterial species and/or AMR.

## 4. Discussion

This study analyzed 112 intestinal samples from wild birds that died naturally or were humanely euthanized at the wildlife rescue center of Vanzago (Italy), to assess the presence and antimicrobial resistance of Gram-negative bacteria, including antimicrobials critical to human medicine. Bacteriological analysis resulted in the isolation of a wide range of bacterial species, many of which are recognized as human pathogens [53], with AST revealing a broad distribution of resistance to several of the tested antimicrobial agents.

Among *Enterobacterales*, *Klebsiella pneumoniae* and *Enterobacter* spp. were identified, both members of the ‘ESKAPE’ group. This acronym refers to a group of highly virulent and AMR pathogens known for their role in nosocomial infections and associated with increased mortality in humans [54,55]. *K. pneumoniae* was isolated in 10 out of 112 (8.9%) wild birds, including both synanthropic and migratory species. The tendency of many wild birds to inhabit urbanized areas is considered a major factor in their exposure to environmental bacteria, including enteric pathogens, thereby increasing the risk of infection and zoonotic transmission [56]. Population density is another influential factor in bacterial circulation; for instance, large aggregations at migratory stopovers or nesting sites facilitate horizontal transmission of pathogens due to frequent interindividual and interspecies contact [56,57]. Furthermore, migration-related stress and immunosuppression may promote both the acquisition and dissemination of infectious agents [53]. Statistical analysis supported these considerations, highlighting that wildland species were less likely to carry major human pathogens (OR = 0.45; *p* = 0.10). Additionally, birds’ age showed a statistical tendency effect on major-pathogen isolation (OR = 0.551, *p* = 0.058), suggesting that adults are less likely than juveniles to carry such pathogens. This finding is consistent with previous studies identifying age as a predictor of the presence of human pathogens and antimicrobial resistance, with younger animals (particularly juvenile gulls) shedding higher proportions of resistant bacteria, likely due to age-related differences in diet and exposure and gut microbiota composition [58,59,60]. Nevertheless, data concerning other avian species are lacking in the literature, highlighting the need for further research on this topic to better understand the role of juvenile wild birds in the dissemination of ARB.

Regarding the AMR profile of *K. pneumoniae* isolates, the highest resistance rates were observed against piperacillin (*n* = 5/10, 50%), ciprofloxacin (*n* = 4/10, 40%), tetracycline (*n* = 4/10, 40%), and sulfamethoxazole/trimethoprim (*n* = 3/10, 30%), followed by piperacillin/tazobactam (*n* = 2/10, 20%). These results partially align with previous studies reporting a general trend of resistance to tetracyclines, sulfonamides, aminoglycosides, phenicols, and fluoroquinolones [61,62,63]. Additionally, four of the *K. pneumoniae* isolates (*n* = 4/10, 40%), from an Alpine Swift (*Tachymarptis melba)*, a European pied Flycatcher (*Ficedula hypoleuca*), a Common Redstart (*Phoenicurus phoenicurus*), and a Common Blackbird (*Turdus merula*), exhibited resistance to third-generation cephalosporins and aztreonam. These isolates also demonstrated resistance to cefepime, a fourth-generation broad-spectrum cephalosporin that has been employed in the treatment of severe infections caused by ESBL-producing Gram-negative bacteria [64]. Notably, 75% (*n* = 3/4) of the cESBL-producing *K. pneumoniae* isolates also displayed a MDR profile. Our phenotypic-only results are consistent with previous studies reporting ESBL-producing and MDR *K. pneumoniae* in wild birds [50,63,65], underscoring the potential role of wild avifauna in the dissemination of ARB of critical importance to human health. Indeed, all antimicrobial agents to which the *K. pneumoniae* isolates showed resistance in this study are classified as medically important for human medicine [12]. In particular, broad-spectrum beta-lactam antibiotics are among the most essential classes of antimicrobials for human use, while piperacillin and piperacillin/tazobactam are authorized exclusively for human use [12]. Furthermore, in the WHO’s most recent update (2024), third-generation cephalosporin-resistant *Enterobacterales* (3GCRE) were included in the “critical group” of the Bacterial Priority Pathogens List, owing to their public health risk, limited therapeutic options, and high disease burden. Specifically, 3GCRE-*K. pneumoniae* poses a particular threat to pediatric populations, complicating first-line antimicrobial treatment, contributing to neonatal sepsis, and being associated with increased morbidity and mortality, particularly in low- and middle-income countries [66].

*Enterobacter* species are well-documented in avian hosts but are generally not associated with disease outbreaks in birds [56]. In this study, the identified species included *Enterobacter hormaechei*, isolated from five out of 112 sampled wild birds (4.5%), and *Enterobacter roggenkampii*, recovered from a single European Serin (*Serinus serinus*) (0.9%). With regard to antimicrobial resistance profiles, none of the *E. hormaechei* isolates exhibited acquired resistance to the tested antimicrobials, apart from resistance to cefoxitin, ampicillin, and amoxicillin/clavulanic acid. These findings are attributable to IR mechanisms, as outlined by CLSI and EUCAST guidelines [30,32]. Conversely, the *E. roggenkampii* isolate displayed MDR phenotype, showing resistance to tetracyclines, sulfonamides, and phenicols. Currently, there is limited direct evidence of *E. hormaechei* and *E. roggenkampii* isolation from wild birds, which hampers direct comparison with the existing literature. However, *E. hormaechei* has been documented in other wildlife species, including wild boars [67], suggesting that various wild animals may serve as reservoirs. The isolation of these species, both members of the *Enterobacter cloacae* complex (ECC) [68,69] (a group of phylogenetically related bacteria known for their pathogenicity and ability to harbor ARGs) from bird species represents an important finding. It underscores the need to include wild birds in environmental surveillance efforts aimed at understanding the circulation and dissemination of pathogenic and resistant bacteria in natural ecosystems.

*Salmonella* spp., a zoonotic bacterium of significant public health concern with notable economic impact [70,71], was detected in 11 out of 112 (9.8%) samples, primarily from raptors and scavenger bird species. Regarding the bird species from which *Salmonella* spp. were isolated, our findings are consistent with the existing literature. Indeed, several studies report that salmonellosis in wild birds is more commonly observed in carnivorous or omnivorous species, as these birds, either by preying on other birds or scavenging in landfills, are at greater risk of consuming *Salmonella*-infected preys or contaminated food sources [72]. Logistic regression confirmed feeding behavior as a strong predictor (OR = 0.142, *p* = 0.004), indicating that granivorous diets are protective compared to carnivorous/scavenger diets. Moreover, habitat (synanthropic vs. wildland species) showed a strong trend towards significance (OR = 12.285, *p* = 0.063), suggesting a much higher risk of *Salmonella* spp. positivity in anthropized environments. In terms of prevalence, the rate identified in our study is comparable to those reported by Molina-López et al. [70] in Catalonia and Millán et al. [73] in Basque Country, who observed isolation rates of 10% (*n* = 12/121) and 8.5% (*n* = 7/82), respectively. Our findings, however, reveal a considerably higher prevalence than that reported by Botti et al. [71], who quoted in north-western Italy a prevalence of 2.2%, (*n* = 24/1101) with half of the isolates recovered from birds of prey.

Alongside *Salmonella* spp., another noteworthy member of the *Enterobacterales* order is *Escherichia coli*. In the present study, *E. coli* accounted for 42% (*n* = 66/157) of all bacterial isolates and were detected in the majority of birds sampled (*n* = 66/112, 59%). With regard to AMR profiles, the highest resistance rates were observed against piperacillin (*n* = 28/66, 42%), ampicillin (*n* = 27/66, 40.9%), and tetracycline (*n* = 22/66, 33%). These findings are consistent with previous studies reporting relatively high levels of resistance to tetracycline and ampicillin among *E. coli* isolated from a wide variety of wild bird species across different geographical regions [74,75,76,77]. Such high resistance rates likely result from the historical and widespread use of these antimicrobials in both human and veterinary medicine [78,79], raising concern given that tetracyclines and penicillins are categorized as “Highly Important Antimicrobials” (HIAs) for human healthcare and are also critical for treating various infections in animals [12,80]. In relation to piperacillin resistance, there is a limited number of studies evaluating this molecule in *E. coli* from wild birds. Nevertheless, the prevalence observed in this study is substantially higher than the 12.0% (*n* = 6/50) and 12.5% (*n* = 4/32) resistance rates reported by Shobrak et al. [81] in non-migratory and migratory birds in Saudi Arabia, respectively, yet lower than the 84.2% (*n* = 48/57) reported by Yuan et al. [79] among migratory birds in the Pearl River Basin, China, likely reflecting variations in environmental antimicrobial exposure, geographic factors, and methodological differences. Additional resistance profiles observed in our *E. coli* isolates included fluoroquinolones and phenicols. Specifically, three isolates (*n* = 3/66; 4.5%) were resistant to both ciprofloxacin and levofloxacin, while two exhibited resistances to ciprofloxacin and intermediate susceptibility to levofloxacin. Four isolates (*n* = 4/66; 6%) from migratory (*Phylloscopus collybita*, *Apus apus*), carnivorous (*Buteo buteo*), and scavenger (*Corvus cornix*) birds were resistant to chloramphenicol. Although these prevalence values are not as high as those reported in other studies across different bird species and regions [79,82,83,84], they nonetheless underscore the importance of AMR surveillance in wildlife. In fact, fluoroquinolones are classified by the WHO as HPCIAs for human health, and chloramphenicol, though permitted for pets, has been banned for use in food-producing animals in the EU since 1994 [12,85,86]. Moreover, one *E. coli* isolated from a Long-eared Owl (*Asio otus*) exhibited resistance to aztreonam, while two other isolates, from a Common Starling (*Sturnus vulgaris*) and a Mallard (*Anas platyrhynchos*), were intermediate-resistant to this last-line agent, which is exclusively authorized for human use. These findings, particularly the association of certain AMR phenotypes with avian ecological groups, lend further support to the hypothesis that environmental exposures and ecological or feeding behaviors significantly influence the likelihood of birds acquiring and disseminating AMR bacteria [15,17,18]. In fact, migratory movement significantly increased odds of piperacillin resistance (OR = 0.282, *p* = 0.048), implicating migratory species as long-range disseminators of AMR.

In this study, 51.5% (*n* = 34/66) of the *E. coli* isolates were resistant to at least one of the antimicrobials tested, and 18.2% (*n* = 12/66) exhibited a MDR profile. None of the *E. coli* isolates showed a cESBL-producing profile. Numerous studies have explored the prevalence of ESBL-producing *E. coli* in bird species, particularly those living in close proximity to human populations and feeding on anthropogenic waste, such as gulls and corvids [87,88]. Given that most birds sampled in our study were small passerines and forest-dwelling species, it is important to note that limited data are available on ESBL-producing *E. coli* in these avifauna, with many studies reporting an absence [53,83,89,90]. The absence of cESBL-producing *E. coli* in our study could be explained by the fact that forest species tend to inhabit less anthropized environments and that their diets, primarily based on aerial insects, soft fruits, and berries, reduce the risk of ingesting contaminated food. Since ESBL-producing bacteria are predominantly transmitted via the fecal–oral route, such feeding behaviors may lower exposure risk [88,89]. Notably, no ESBL-producing strains were detected, even in bird species previously associated with such findings, including Common Blackbirds (*Turdus merula*) [91], gulls [92], corvids [93] and raptors [76]. This highlights the importance of long-term surveillance, using, eventually, individually marked birds, as emphasized by Järhult et al. [70], to better understand the environmental dynamics of AMR bacteria.

Other bacterial species isolated from the sampled animals included *Proteus* spp. (*n* = 34/157, 21.7%), *Citrobacter* spp. (*n* = 4/157, 2.5%), *Hafnia* spp. (*n* = 4/157, 2.5%), *Morganella* spp. (*n* = 3/157, 1.9%), and *Serratia* spp. (*n* = 2/157, 1.3%). These taxa are commonly isolated from wild birds and are recognized as opportunistic pathogens in humans [48,53,83,94]. Across various studies, the most consistently reported resistance among these genera was to ampicillin, observed in nearly all isolates, regardless of host species or geographical location [53,83,95,96]. Resistance to other antimicrobials, such as tetracyclines, was also frequently reported [53,83,95,96]. These findings likely reflect a combination of IR mechanisms and selective environmental pressures. Notably, in this study, out of nine *Enterobacterales* isolates (*n* = 9/157, 6.1%) resistant to tigecycline, four were identified as *Proteus mirabilis*, and three as *Morganella morganii*. Tigecycline, a glycylcycline antimicrobial derived from tetracyclines, is considered a last-resort antimicrobial agent for the treatment of severe infections caused by MDR bacterial strains [97]. Therefore, the detection of resistance to tigecycline is a cause for concern in the current context of rising AMR. In this regard, it is important to note that EUCAST has classified tigecycline as having insufficient activity against certain species, including *Klebsiella* spp., *Proteus* spp., *Morganella* spp., and *Providencia* spp. [98,99]. This intrinsic reduced susceptibility may partially explain the resistance profiles observed in the present study for these specific genera and species.

Within the order *Pseudomonadales*, *P. aeruginosa* and *A. baumannii* were isolated from 4.5% (*n* = 5/112) and 0.9% (*n* = 1/112) of the sampled wild birds, respectively. These findings are consistent with previous studies conducted on wild bird populations across various countries, particularly in terms of the *A. baumannii* isolation rate. For example, Ahmed et al. [48] reported *P. aeruginosa* prevalence rates of 10.0% (*n* = 8/80) and 18.3% (*n* = 11/60) in resident and migratory birds in Egypt, respectively, whereas Rodrigues et al. [100] detected *P. aeruginosa* in only 1 of 115 fecal samples from wild birds in England. A study by Russo et al. [101] in southern Italy reported a 36% prevalence of *P. aeruginosa* from cloacal swabs of 163 wild birds, yet no *A. baumannii* were isolated. Conversely, in Nigeria, Dahiru et al. [102] reported *A. baumannii* in 31.3% (*n* = 15/48) of fecal samples from wild birds, while in Ukraine, Łopińska et al. [103] detected only two *A. baumannii* isolates among 1051 fecal samples. Regarding AST results, both *P. aeruginosa* and *A. baumannii* isolates in the present study exhibited high susceptibility rates to the tested antimicrobials. All *P. aeruginosa* isolates were susceptible to the antimicrobials tested, while the *A. baumannii* isolate showed resistance to cefotaxime and intermediate resistance to ceftazidime and cefepime. The AST results for *P. aeruginosa* are consistent with those of Russo et al. [100], though they differ from studies reporting higher resistance levels [100,104]. The reduced susceptibility of *A. baumannii* to cephalosporins observed in this study is corroborated by the findings of Łopińska et al. [103], and can be attributed to the organism’s IR mechanisms, including the production of chromosomally encoded cephalosporinases and other resistance determinants [103,105,106,107]. Given this, it is important to highlight that for these bacteria species, the evaluation of the bacterial resistance/susceptibility for several antimicrobials was not possible due to the lack of defined breakpoints. Both *P. aeruginosa* and *A. baumannii* are ESKAPE pathogens with high clinical relevance but are rarely part of the normal microbiota. *P. aeruginosa* is typically environmental, while the reservoir of *A. baumannii* is unclear [103,108,109]. They are major causes of hospital infections, with the WHO listing *A. baumannii* as critical and *P. aeruginosa* as high priority in 2024 [66]. Their intrinsic and acquired resistance limits treatment options [100,103,105,110], and their detection in wildlife highlights potential environmental and zoonotic roles [108].

*Acinetobacter pittii* is a member of the *Acinetobacter calcoaceticus–baumannii* (ACB) complex. Although associated with nosocomial infections, this bacterium is often overlooked due to its relatively lower rates of AMR and detection compared to *A. baumannii*. However, emerging evidence indicates that MDR *A. pittii* are increasingly involved in hospital-acquired infections, leading to growing recognition of this pathogen’s clinical importance [111,112]. To date, information regarding the genetic background and virulence determinants of *A. pittii* remains limited and is often extrapolated from *A. baumannii* due to their high genomic similarity [112]. Nevertheless, recent studies have shown that, similarly to *A. baumannii*, *A. pittii* exhibits natural low susceptibility to narrow-spectrum beta-lactam antibiotics, and, although less frequently, may acquire ESBLs-genes through horizontal gene transfer mechanisms [111,112,113]. In the present study, a single *A. pittii* isolate obtained from a Long-eared Owl (*Asio otus*) demonstrated resistance to cefotaxime and intermediate resistance to ceftazidime and cefepime. Additionally, the isolate was resistant to piperacillin and showed intermediate resistance to piperacillin–tazobactam. Given its positivity in the DDST and growth on selective medium for ESBL-producing bacteria, this AMR profile may be indicative of ESBL production. Although this is not the first reported isolation of *A. pittii* from wild birds [103,114], our findings reinforce the need for broader surveillance of AMR emerging pathogens in non-clinical environments.

Another interesting finding of this study was the isolation of *Achromobacter mucicolens* from a Eurasian Collared Dove (*Streptopelia decaocto*). This species, part of the *Burkholderiales* order, is an emerging opportunistic pathogen in immunocompromised individuals, associated with pneumonia, meningitis, and sepsis [37,38]. *Achromobacter* spp. are IR to many antimicrobials, though resistance mechanisms remain incompletely understood [37,39,115,116]. Two multi-drug efflux pumps and chromosomally encoded beta-lactamases (e.g., OXA-114 and AZM-11) have been linked to this IR [37,39,115,116,117]. While OXA-114 shows in vitro activity against early-generation cephalosporins and piperacillin, its clinical impact remains unclear [39,118]. *Achromobacter* spp. can also acquire ARGs, contributing to MDR phenotypes and limiting treatment options [119]. Despite their clinical relevance, susceptibility testing is complicated by the lack of CLSI breakpoints. EUCAST provides breakpoints only for *A. xylosoxidans* for a few agents, including piperacillin–tazobactam and meropenem [30,34]. In this study, AST interpretation for *A. mucicolens* was possible by integrating EUCAST guidelines with data from Almuzara et al. [38]. The isolate was susceptible to piperacillin–tazobactam, carbapenems, tetracyclines, and sulfonamides. Large inhibition zones were also observed for tigecycline and piperacillin, consistent with previous studies [38,39,116,120]. Resistance to aztreonam and cefoxitin, and intermediate susceptibility to gentamicin, aligned with published profiles of clinical isolates [39].

In this study, 20 Gram-negative isolates exhibited resistance phenotypes compatible with AmpC production. These included five *P. aeruginosa*, five *E. hormaechei*, three *M. morganii*, two *C. braakii*, two *C. freundii*, one *K. aerogenes*, one *E. roggenkampii*, and one *L. amnigena* isolate. AmpC beta-lactamases are clinically significant cephalosporinases, typically chromosomally encoded in many *Enterobacteriaceae* and some non-fermenters like *P. aeruginosa*. These enzymes confer resistance to cefoxitin, first-generation cephalosporins, most penicillins, and beta-lactam/beta-lactamase inhibitor combinations [106,109,121]. In many bacterial species, AmpC expression is inducible and may be upregulated through mutations, potentially leading to resistance against extended-spectrum cephalosporins and subsequent treatment failure. Consequently, third-generation cephalosporins, although potentially active in vitro, should generally be avoided for treating infections caused by wild-type-inducible AmpC producers, with alternative agents, such as piperacillin, piperacillin–tazobactam, and tigecycline, being preferred [106,121,122,123]. In this regard, all *Enterobacterales* isolates with a cAmpC profile in the present study belong to species recognized in the literature as chromosomally encoded AmpC producers [106,121,122]. Their AMR profiles are also consistent with an AmpC-producing profile, as they exhibited resistance to cefoxitin, ampicillin, and amoxicillin–clavulanic acid, while remaining susceptible to third-generation cephalosporins and cefepime. Notably, the two *C. freundii* isolates were resistant to piperacillin, while *M. morganii* isolates showed resistance to tigecycline. Regarding *L. amnigena*, previously known as *Enterobacter amnigenus*, this bacterium is recognized to be an unusual opportunistic human pathogen [124]. The natural resistance of *L. amnigena* to second- and third-generation cephalosporins, as well as to other antimicrobials, such as gentamicin and doxycycline, is described in the literature [125,126], supporting our results. However, contradictory result had been reported [127]. To date, several studies have reported that *L. amnigena* possesses the *ampC* gene; however, no detailed studies on the *L. amnigena* AmpC beta-lactamase have been documented, though a new chromosomally-encoded AmpC beta-lactamase, *bla*LAQ-1, was recently described [124,127]. Although *Hafnia alvei*, *Providencia* spp., and *Serratia marcescens* are also recognized in the literature as natural AmpC producers [106,121,122], none of the isolates from these species in our study displayed cAmpC phenotypes or resistance to cefoxitin. In these genera, AmpC expression is typically low and inducible, and its phenotypic manifestation depends on the presence of mutations in regulatory genes or environmental pressure. In the absence of such factors, AmpC expression may remain below detection thresholds used in phenotypic tests [128]. Furthermore, methodological limitations of the phenotypic assays used may also have contributed to false-negative results [121]. These findings align with other reports where species known to carry inducible AmpC did not always exhibit phenotypic activity or cefoxitin resistance [121,123,129].

Finally, in the present study, 4% (*n* = 6/157) of the bacterial isolates tested were found to be resistant to colistin; of these, four were *E. coli* and two were *K. pneumoniae*. Colistin has been widely used since the mid-20th century in veterinary medicine, particularly in swine, for the treatment of enteric infections [130,131]. Today, it is regarded as a last-resort antimicrobial for managing human infections caused by MDR Gram-negative bacteria, including carbapenemase-producing *Enterobacterales*, *Acinetobacter baumannii*, and *Pseudomonas aeruginosa* [132,133,134]. Recognizing its important role, the WHO has classified colistin among the HPCIA antimicrobials [12]. Reflecting similar concerns, the EMA has placed colistin in Category B (“Restrict”) of its antimicrobial classification system, recommending that its use in animals be strictly limited to safeguard public health [13]. Since its initial discovery in China in 2015, the plasmid-mediated *mcr-1* gene conferring resistance to colistin has been detected globally in *Enterobacteriaceae* isolates originating from food-producing animals, companion animals, food products, environmental samples, and human clinical cases [135,136]. Similar findings to our study have been reported in wild birds from various parts of the world, highlighting the role of wild birds as potential vectors of colistin-resistant bacteria. For instance, studies have identified *mcr*-positive *Escherichia coli* in gulls, egrets, and other avian species frequenting urban, coastal, and agricultural environments [48,51,137,138]. These birds likely acquire resistant strains through contact with contaminated water, soil, or anthropogenic waste, thereby contributing to the environmental dissemination of mobile colistin resistance genes [48,51,137,138]. These results emphasize the importance of including wildlife, especially birds, in One Health surveillance strategies targeting critical resistance threats.

This study provides important preliminary insights into the distribution of AMR bacteria in wild bird populations, encompassing both classical human pathogens and lesser-known bacterial species. Nonetheless, several limitations must be acknowledged. First, *Salmonella* isolates were not serotyped, as none exhibited resistance profiles of particular relevance. Given the study’s focus on AMR rather than pathogenicity per se, serotyping was deemed beyond its scope. However, given the well-known differences in zoonotic potential among *Salmonella* serotypes, further in-depth investigations are warranted. From this perspective, the preliminary results of this study, including statistical data, isolate counts, and AMR profiles, can provide a valuable foundation for refining the sampling strategy toward a more targeted and ecologically balanced approach. These findings may also guide the development of a more tailored antimicrobial testing panel for *Salmonella*, including compounds not assessed here and agents commonly used in human clinical settings, with the final aim of better evaluating the public health risks posed by AMR *Salmonella* isolates of zoonotic significance. Second, as the findings are based on phenotypic AST, isolates exhibiting phenotypically compatible ESBL or AmpC profiles cannot be definitively classified as enzyme producers, and the potential for false negatives remains. In fact, while the results of phenotypic AST remain valuable, limitations must be acknowledged. These include the inability to detect silent or non-expressed resistance genes, as well as the risk of misclassifying isolates due to overlapping phenotypic profiles or low-level expression of resistance mechanisms. Phenotypic tests may also fail to identify emerging or uncommon resistance genes, particularly in bacterial taxa that are underrepresented in current AMR databases [128,139,140]. Nonetheless, considering these limitations, the importance of genotypic characterization in AMR surveillance remains unquestionable. Despite this, the primary objective of this study was to provide a broad phenotypic overview of resistance patterns in Gram-negative bacteria isolated from wild birds. Given the exploratory nature of the work, the diversity of bacterial and host species, the complexity of potential resistance mechanisms (both plasmidic and chromosomal), the limited availability of genomic reference data for several poorly characterized bacterial species, and the resource constraints typical of broad-scale studies, extensive genotypic analyses were not feasible at this stage. Such investigations would require a dedicated and more focused study. Nevertheless, the present findings offer a meaningful baseline for future hypothesis-driven molecular research, with a potential focus on ARGs and ARB of highest public health relevance. A further limitation of the present study concerns the heterogeneity of bird species sampled, which complicates efforts to establish robust associations between host species and specific resistance profiles or bacterial taxa. Despite this, statistically significant associations were observed, for example, the isolation of *Salmonella* spp. in carnivorous birds and of piperacillin-resistant *E. coli* in migratory species, suggesting that certain avian groups may act as important carriers or amplifiers of AMR bacteria. These findings support the inclusion of specific bird categories in targeted surveillance protocols. An additional point worth mentioning concerns the potential sampling bias associated with the use of fecal samples from wild birds that died at a wildlife rescue center. These birds typically present, upon admission, a compromised health status, which may influence the microbiological profiles characteristic of free-ranging bird populations. Conditions such as stress and immunosuppression may have affected both the prevalence and shedding of ARB in these individuals; given this, the methodological decision to include only birds that had not received antimicrobial treatment prior to death was intended to approximate, as closely as possible, the microbiological condition reflective of the animals’ free-ranging lives. Moreover, it is important to emphasize that, despite this potential bias, wildlife rescue facilities can provide a valuable and ethical opportunity to monitor AMR in wild species, offering access to a wide range of avian taxa that would otherwise be difficult to sample under natural conditions. Among the key strengths of the study is its comprehensive ecological scope, which includes both common and rarely reported bacterial taxa and a wide array of avian hosts. In particular, although certain bacterial species, such as *Acinetobacter baumannii*, *Acinetobacter pittii*, *Pseudomonas aeruginosa*, and *Achromobacter mucicolens,* were isolated at low frequencies, their identification is nonetheless epidemiologically significant. These bacteria are globally recognized for their intrinsic and acquired resistance mechanisms, particularly against last-resort antimicrobials, and are listed among the highest priority pathogens by the WHO due to their limited therapeutic options and association with severe nosocomial infections [37,66,105,111]. Their detection in wild birds, especially in species that interact with anthropogenic environments, raises concern over the potential role of these birds as environmental reservoirs and long-distance spreaders of high-risk AMR determinants, as well as relevant pathogens. Moreover, the recovery of phenotypically colistin-resistant *E. coli* and *K. pneumoniae*, despite being limited in number, further underscores the public health relevance of such findings, considering the role of colistin as a last-resort treatment in human medicine [134]. The detection of these low-frequency but high-risk isolates highlights the importance of continued AMR surveillance in wildlife, as even rare detection events may reflect broader environmental circulation and emerging zoonotic threats, posing a considerable public health threat. This broad approach enhances our understanding of the AMR landscape in wild birds and emphasizes their relevance as sentinels in environmental and public health monitoring frameworks.

## 5. Conclusions

The findings of this study underscore the role of wild birds in the dissemination of AMR Gram-negative microorganisms of concern to human health, including *Klebsiella pneumoniae*, *Acinetobacter baumannii*, and *Enterobacter* spp. The identification of MDR isolates, as well as isolates with resistance phenotypes compatible with the production of ESBL and AmpC, highlights the contribution of avian wildlife to the broader environmental resistome. Despite the natural absence of direct antimicrobial exposure in wild birds, the detection of resistance to HPCIAs, CIAs, and compounds used exclusively in human medicine suggests a contamination scenario shaped by anthropogenic pressures. The proximity of avifauna to urban areas, wastewater effluents, landfills, and livestock production sites likely facilitates the environmental transmission of resistance determinants to wild bird populations [15,75]. Moreover, observed differences in resistance patterns across ecological guilds further support the influence of dietary habits and habitat use on AMR acquisition [141]. Migratory species may additionally function as effective bioindicators of the environmental AMR burden and serve as long-distance vectors for resistant bacteria, underscoring their relevance within a One Health surveillance framework [17,142]. The detection of zoonotic and opportunistic human pathogens carrying complex resistance traits in wild birds is therefore of considerable concern for both animal and human health, highlighting the urgent need for integrated, cross-sectoral monitoring strategies [142]. To effectively address the AMR crisis, wildlife—especially birds—should be more explicitly incorporated into global surveillance strategies. Doing so would enable better understanding of transmission dynamics and support the development of targeted interventions aimed at curbing the environmental spread of resistance.

## Figures and Tables

**Table 1:** Classification of sampled avian species.

Taxonomic Order	Family	Scientific Name	Common Name	Total
*Accipitriformes*	*Accipitridae*	*Buteo buteo*	Eurasian Buzzard	1
*Anseriformes*	*Anatidae*	*Anas platyrhynchos*	Mallard	7
*Apodiformes*	*Apodidae*	*Tachymarptis melba*	Alpine Swift	4
		*Apus apus*	Common Swift	10
*Bucerotiformes*	*Upupidae*	*Upupa epops*	Eurasian Hoopoe	1
*Charadriiformes*	*Laridae*	*Larus michahellis*	Yellow-legged Gull	1
	*Scolopacidae*	*Scolopax rusticola*	Eurasian Woodcock	1
*Columbiformes*	*Columbidae*	*Columba palumbus*	Common Woodpigeon	8
		*Streptopelia decaocto*	Eurasian Collared Dove	10
*Coraciiformes*	*Alcedinidae*	*Alcedo atthis*	Eurasian Kingfisher	2
*Falconiformes*	*Falconidae*	*Falco tinnunculus*	Common Kestrel	8
		*Falco peregrinus*	Peregrine Falcon	1
*Gruiformes*	*Rallidae*	*Gallinula chloropus*	Common Moorhen	1
*Passeriformes*	*Corvidae*	*Corvus cornix*	Hooded Crow	4
		*Pica pica*	Eurasian Magpie	6
		*Garrulus glandarius*	Eurasian Jay	1
	*Fringillidae*	*Fringilla coelebs*	Eurasian Chaffinch	1
		*Serinus serinus*	European Serin	4
	*Hirundinidae*	*Hirundo rustica*	Barn Swallow	3
		*Delichon urbicum*	Common house Martin	1
	*Muscicapidae*	*Ficedula hypoleuca*	European pied Flycatcher	1
		*Phoenicurus phoenicurus*	Common Redstart	1
		*Erithacus rubecula*	European Robin	3
	*Paridae*	*Parus major*	Great Tit	2
	*Phylloscopidae*	*Phylloscopus collybita*	Common Chiffchaff	1
	*Regulidae*	*Regulus regulus*	Goldcrest	1
	*Sturnidae*	*Sturnus vulgaris*	Common Starling	1
	*Sylviidae*	*Sylvia atricapilla*	Blackcap	1
	*Turdidae*	*Turdus merula*	Common Blackbird	10
*Pelecaniformes*	*Ardeidae*	*Ardea cinerea*	Grey Heron	1
		*Bubulcus ibis*	Cattle Egret	1
		*Egretta garzetta*	Little Egret	1
*Piciformes*	*Picidae*	*Picus viridis*	Green Woodpecker	1
*Strigiformes*	*Strigidae*	*Strix aluco*	Tawny Owl	1
		*Otus scops*	Eurasian Scops-owl	2
		*Athene noctua*	Little Owl	8
		*Asio otus*	Long-eared Owl	1
Total				112

## Data Availability

The original contributions presented in the study are included in the article; further inquiries can be directed to the corresponding author.

## Supplementary Materials

The following supporting information can be downloaded at: https://www.mdpi.com/article/10.3390/ani15152289/s1, Table S1: AST interpretive criteria used for Gram-negative bacterial isolates recovered from wild birds; Figure S1: Radial tree graph showing the distribution of 157 Gram-negative bacterial strains isolated from 112 wild bird specimens; Table S2: Distribution of Gram-negative bacterial isolates, grouped by genus, across wild bird species sampled; Table S3: Antimicrobial resistance profile of *Enterobacterales* isolates; Table S4: Antimicrobial resistance profile of *Pseudomonadales* isolates; Table S5: Antimicrobial resistance profile of *Burkholderiales* isolates; Table S6: Antimicrobial susceptibility (Kirby–Bauer method), number and percentage of resistant strains by antimicrobial agent and bacterial species within *Enterobacterales* order; Table S7: Antimicrobial susceptibility (Kirby–Bauer method), number and percentage of resistant strains by antimicrobial agent and bacterial species within *Pseudomonadales* order; Table S8: Antimicrobial susceptibility (Kirby–Bauer method), number and percentage of resistant strains by antimicrobial agent and bacterial species within *Burkholderiales* order; Table S9: Binary logistic regression for major pathogen isolation; Table S10: Binary logistic regression for *Salmonella* spp. isolation; Table S11: Binary logistic regression for piperacillin resistance in *E. coli*.
