# Supplementary material for: Resisting the Final Line: Phenotypic Detection of Resistance to Last-Resort Antimicrobials in Gram-Negative Bacteria Isolated from Wild Birds in Northern Italy
Source: Animals (Basel). 2025 Aug 5;15(15):2289. doi: 10.3390/ani15152289 (PMC12345454; doi:10.3390/ani15152289)
Supplement: Supplementary file 1 [file animals-15-02289-s001.zip › animals-3768993-supplementary.pdf]

Table S1. AST interpretive criteria used for Gram-negative bacterial isolates recovered from wild birds. This table summarizes the zone diameter breakpoints (mm) and MIC values (µg/mL) used for interpreting phenotypic AST results across different bacterial taxa, including *Enterobacterales*, *Pseudomonadales*, and *Burkholderiales*. Breakpoints were primarily derived from EUCAST (version 15.0 and 14.0) and CLSI (M100, 34th and 35th editions) guidelines. In cases where no official breakpoints were available, alternative criteria were applied, as reported in the legend. As no breakpoints were available for *Comamonas* spp., and no reference values could be found in the literature for this species or closely related bacteria, this isolate was not included in the breakpoint definitions provided in this table.

			Enterobacterales				*Pseudomonadales*							*Burkholderiales*		
							Acinetobacter spp.			*Pseudomonas aeruginosa*				*Achromobacter* spp.		
			**Interpretive Categories and Zone Diameter Breakpoints (mm)**				**Interpretive Categories and Zone Diameter Breakpoints (mm)**			**Interpretive Categories and Zone Diameter Breakpoints (mm)**				**Interpretive Categories and Zone Diameter Breakpoints (mm)**		
**Antimicrobial classes**	**Molecules**	**Disk content (µg)**	**S**	**I**	**R**	**Disk content (µg)**	**R**	**I**	**S**	**R**	**I**	**S**	**Disk content (µg)**	**R**	**I**	**S**
Aminoglycosides	CN	10	≥ 17[1]	-	< 17[1]	10	≥ 17[1]	-	< 17[1]	≥ 13[5]	-	< 13[5]	10	≥ 21[L]	20-16[L]	≤ 15[L]
Cephalosporins	CTX	5	≥ 20[1]	19-17[1]	< 17[1]	30	≥ 23[2c]	22-15[2c]	≤ 14[2c]	NB			5	NB		
	CAZ	10	≥ 22[1]	21-19[1]	< 19[1]	30	≥ 18[2c]	17-15[2c]	≤ 14[2c]	≥ 18[2d]	17-15[2d]	≤ 14[2d]	10	NB		
	FEP	30	≥ 27[1]	26-24[1]	< 24[1]	30	≥ 18[2c]	17-15[2c]	≤ 14[2c]	≥ 18[2d]	17-15[2d]	≤ 14[2d]	30	NB		
	CZA	10/4	≥ 13[1]	-	< 13[1]	10/4	NB			≥ 17[1]	-	< 17[1]	10/4	NB		
Cephamycins	FOX	30	≥ 18[2a,§]	17-15[2a,§]	≤ 14[2a,§]	/	NT			NT			30	NB		
Tetracyclines	TE	30	≥ 15[2a,2b]	12-14[2a,2b]	≤ 11[2a,2b]	30	NB			NB			30	≥ 29[L]	28-21[L]	≤ 20[L]
	DO	30	≥ 14[2a,2b]	11-13[2a,2b]	≤ 10[2a,2b]	30	NB			NB			30	≥ 24[L]	23-21[L]	≤ 20[L]
Glycylcyclines	TGC	15	≥ 18[1,*]	-	< 18[1,*]	15	NB			NB			15	NB		
Fluoroquinolones	LEV	5	≥ 23[1]	22-19[1]	< 19[1]	5	≥ 23[1]	22-20[1]	< 20[1]	≥ 22[2d]	21-15[2d]	≤ 14[2d]	5	NB		

	CIP	5	≥ 25[1]	24-22[1]	< 22[1]	5	≥ 50[1]	49-21[1]	< 21[1]	≥ 25[2d]	24-19[2d]	≤ 18[2d]	5	NB		
			Salmonella spp. only													
			≥ 31[2b]	30-21[2b]	≤ 20[2b]											
Carbapenems	MEM	10	≥ 22[1]	21-16[1]	< 16[1]	10	≥ 21[1]	20-15[1]	< 15[1]	≥ 19[2d]	18-16[2d]	≤ 15[2d]	10	≥ 26[1,**]	25-20[1,**]	< 20[1,**]
	IPM	10	≥ 22[1]	21-19[1]	< 19[1]	10	≥ 24[1]	23-21[1]	< 21[1]	≥ 19[2d]	18-16[2d]	≤ 15[2d]	10	≥ 22[L]	21-12[L]	≤ 11[L]
			Morganellaceae only													
			≥ 50[1]	49-19[1]	< 19[1]											
Penicillis	AMP	10	≥ 17[2a,2b]	16-14[2a,2b]	≤ 13[2a,2b]	/	NT			NT			10	NB		
	AMC	20/10	≥ 18[2a,§]	17-14[2a,§]	≤ 13[2a,§]	/	NT			NT			20/10	NB		
	PRL	30	≥ 20[1]	-	< 20[1]	100	≥ 21[2c]	20-18[2c]	≤ 17[2c]	≥ 22[2d]	21-18[2d]	≤ 17[2d]	30	NB		
	TZP	30/6	≥ 20[1]	-	< 20[1]	100/10	≥ 21[2c]	20-18[2c]	≤ 17[2c]	≥ 22[2d]	21-18[2d]	≤ 17[2d]	30/6	≥ 26[1,**]	-	< 26[1,**]
Sulphonamides	SXT	1.25/23.75	≥ 14[1]	13-11[1]	< 11[1]	1.25/23.75	≥ 14[1]	13-11[1]	< 11[1]	NB			1.25/23.75	≥ 26[1,**]	-	< 26[1,**]
Phenicols	C	30	≥ 18[2a,2b]	13-17[2a,2b]	≤ 12[2a,2b]	/	NT			NT			30	NB		
Monobactam	ATM	30	≥ 26[1]	25-21[1]	< 21[1]	30	NB			≥ 22[2d]	21-16[2d]	≤ 15[2d]	30	NB		
			**Interpretive Categories and MIC Breakpoints (μg/mL)**				**Interpretive Categories and MIC Breakpoints (μg/mL)**			**Interpretive Categories and MIC Breakpoints (μg/mL)**				**Interpretive Categories and MIC Breakpoints (μg/mL)**		
**Antimicrobial classes**	**Molecules**	**MIC Test Strip content (μg/mL)**	**S**	**I**	**R**	**MIC Test Strip content (μg/mL)**	**S**	**I**	**R**	**S**	**I**	**R**	**MIC Test Strip content (μg/mL)**	**S**	**I**	**R**
Polymyxin	CS	0.016-256	≥ 2[4]	-	< 2[4]	0.016-256	≥ 2[4]	-	< 2[4]	≥ 4[4]	-	< 4[4]	0.016-256	≥ 8**	-	< 8**

[1] = EUCAST - Breakpoint tables for interpretation of MICs and zone diameters, Version 15.0 and 14.0 [1,2]; [2a] = CLSI M100 - Performance Standards for Antimicrobial Susceptibility Testing, 34th and 35th Edition (Table 2A-1) [3,4]; [2b] = CLSI M100 - Performance Standards for Antimicrobial Susceptibility Testing, 34th and 35th Edition (Table 2A-2) [3,4]; [2c] = CLSI

M100 - Performance Standards for Antimicrobial Susceptibility Testing, 34th and 35th Edition (Table 2B-2) [3,4]; [2d] = CLSI M100 - Performance Standards for Antimicrobial Susceptibility Testing, 34th and 35th Edition (Table 2B-1) [3,4]; [4] = Liofilchem ® - Table no. 1 MTS™ Interpretative Criteria and Quality Control (EUCAST MIC breakpoints) [5]; [5] = EUCAST ECOFF defined for CN in the case of *Pseudomonas aeruginosa;* [L] = The breakpoints used are those reported in the scientific literature [6]; * = Breakpoints reported by EUCAST for *E. coli* and *C. koseri* [1,2]; ** = Breakpoints reported by EUCAST for *Achromobacter xylosoxidans* [7]; [§] = For *Salmonella* spp., the breakpoints reported by CLSI M100 for *Enterobacterales* (Table 2A-1) were used, given the absence of specific breakpoints for this genus in CLSI M100-Table 2A-2; NT = Not Tested; NB = No Breakpoints available in the latest version of CLSI and EUCAST official guidelines (2025) [1,3].

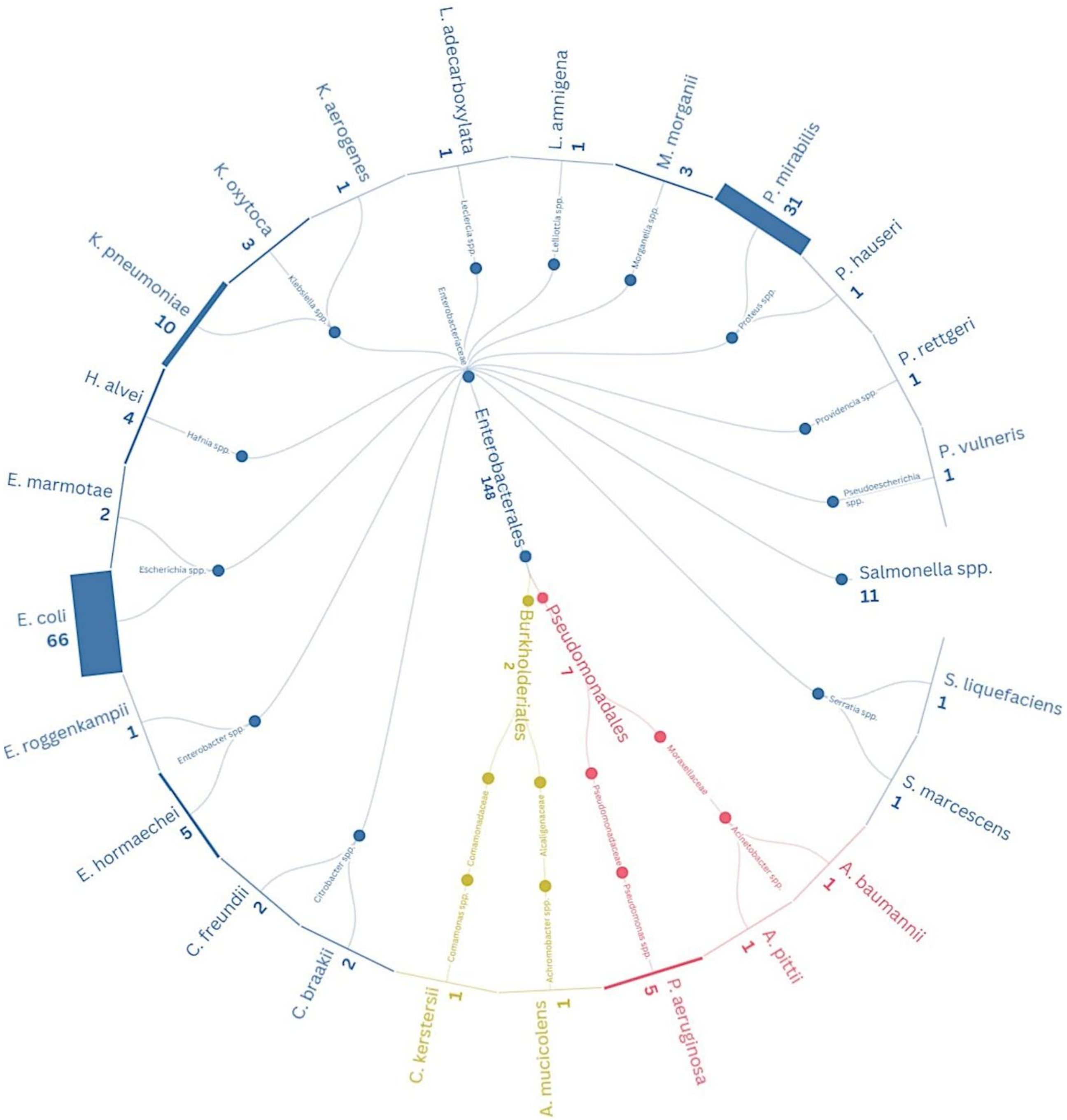

Figure S1. Radial tree graph showing the distribution of 157 Gram-negative bacterial strains isolated from 112 wild bird specimens. Bacteria are grouped by order (central nodes), genus, family, and species (outermost tips). Bar sizes reflect the number of isolates for each bacterial species. The color of each branch represents the bacterial order: blue for *Enterobacterales*, red for *Pseudomonadales*, and yellow for *Burkholderiales*. A genome-scale taxonomic analysis of the entire order *Enterobacterales* recently performed reassigned *Hafnia* spp. to the newly established family *Hafniaceae* (fam. nov.), *Serratia* spp. to *Yersiniaceae* (fam. nov.), and *Morganella*, *Proteus*, and *Providencia* spp. to *Morganellaceae* (fam. nov.) [8]. Nevertheless, these genera have traditionally been classified within the family *Enterobacteriaceae* [9].

Table S2. Distribution of Gram-negative bacterial isolates, grouped by genus, across wild bird species sampled.

**Sampled wild bird species**	**Isolated bacteria (divided per genus)**																
	Achromobacter spp.	*Escherichia* spp.	*Proteus* spp.	*Salmonella* spp.	*Klebsiella* spp.	*Enterobacter* spp.	*Hafnia* spp.	*Morganella* spp.	*Citrobacter* spp.	*Comamonas* spp.	*Leclercia* spp.	*Lelliottia* spp.	*Providencia* spp.	*Pseudoescherichia* spp.	*Serratia* spp.	*Pseudomonas* spp.	*Acinetobacter* spp.
Upupa epops (n=1)		1						1									
Sylvia atricapilla (n=1)		1															
Sturnus vulgaris (n=1)		2															
Strix aluco (n=1)		1															
Scolopax rusticola (n=1)		1					1										
Regulus regulus (n=1)		1															
Picus viridis (n=1)		1					1						1				
Phylloscopus collybita (n=1)		1															
Phoenicurus phoenicurus (n=1)		1	1														
Larus michahellis (n=1)		1															
Garrulus glandarius (n=1)		1															
Gallinula chloropus (n=1)		1	1		1					1							
Fringilla coelebs (n=1)		1				1						1					
Ficedula hypoleuca (n=1)		1															
Falco peregrinus (n=1)		1															
Egretta garzetta (n=1)						1											
Delichon urbicum (n=1)																	

Buteo buteo (n=1)		1															
Bubulcus ibis (n=1)		1	1														
Asio otus (n=1)		1															1
Ardea cinerea (n=1)		1	1	1													
Parus major (n=2)			1													1	
Otus scops (n=2)		2	1	1	1	1											
Alcedo atthis (n=2)																	
Hirundo rustica (n=3)		2	1				1									1	
Erithacus rubecula (n=3)		1															
Tachymarptis melba (n=4)		2	1		3												
Serinus serinus (n=4)		2				1			1							1	
Corvus cornix (n=4)		2	4	1													
Pica pica (n=6)		2	1	3	1	1		1	1								1
Anas platyrhynchos (n=7)		3	1														
Falco tinnunculus (n=8)		4	1	1											2		
Columba palumbus (n=8)		4	1														
Athene noctua (n=8)		7	3	4			1										
Turdus merula (n=10)		9	7		4			1								1	
Streptopelia decaocto (n=10)	1	4	1		1	1					1			1		1	
Apus apus (n=10)		4	5		1				2								

Table S3. Antimicrobial resistance profile of *Enterobacterales* isolates. For all isolated bacterial strains, the interpretation of inhibition zones ("S", "I", "R") is reported based on breakpoints defined by EUCAST and CLSI. In the absence of species-specific breakpoints, those defined for related species or for species within the same taxonomic order were applied. Where no breakpoints were available for the agent and the organism, the measured inhibition zone diameter (in mm) is reported.

**Wild bird species**	**Isolated bacteria strains**	**Antimicrobial susceptibility testing panel**																				
		Aminoglycosides	Cephalosporins				Cephamycins	Tetracyclines		Glycylcyclines	Fluoroquinolones		Carbapenems		Penicillis				Sulphonamides	Phenicols	Monobactam	Polymyxin
		CN	CTX	CAZ	FEP	CZA	FOX	TE	DO	TGC	LEV	CIP	MEM	IPM	AMP	AMC	PRL	TZP	SXT	C	ATM	CS
Apus apus	*Citrobacter braakii* [AmpC]	S	S	S	S	S	R[IR]	S	S	S	S	S	S	S	R[IR]	R[IR]	S	S	S	S	S	S
Pica pica	*Citrobacter braakii* [AmpC]	S	S	S	S	S	R[IR]	S	S	S	S	S	S	S	R[IR]	R[IR]	S	S	S	S	S	S
Apus apus	*Citrobacter freundii* [AmpC; MDR]	S	S	S	S	S	R[IR]	R	I	S	S	S	S	S	R[IR]	R[IR]	R	S	R	R	S	S
Ardea cinerea	*Citrobacter freundii* [AmpC; MDR]	R	S	S	S	S	R[IR]	S	S	S	S	S	S	S	R[IR]	R[IR]	R	S	S	S	R	S
Columba palumbus	*Escherichia coli*	S	S	S	S	S	S	S	S	S	S	S	S	S	S	S	S	S	S	S	S	S
Athene noctua	*Escherichia coli*	S	S	S	S	S	S	S	S	S	S	S	S	S	R	S	R	S	S	S	S	S
Athene noctua	*Escherichia coli*	S	S	S	S	S	S	R	R	S	S	S	S	S	R	S	R	S	S	S	S	S
Falco peregrinus	*Escherichia coli* [MDR]	S	S	S	S	S	S	S	S	S	R	R	S	S	R	S	R	S	R	S	S	S
Larus michahellis	*Escherichia coli* [MDR]	S	S	S	S	S	S	R	I	S	I	R	S	S	R	S	R	S	S	S	S	S
Erithacus rubecula	*Escherichia coli*	S	S	S	S	S	S	S	S	S	S	S	S	S	S	S	S	S	S	S	S	S
Hirundo rustica	*Escherichia coli*	S	S	S	S	S	S	S	S	S	S	S	S	S	S	S	S	S	S	S	S	R
Tachymarptis melba	*Escherichia coli*	S	S	S	S	S	S	S	S	S	S	S	S	S	S	S	S	S	S	S	S	S
Asio otus	*Escherichia coli* [MDR]	R	S	S	S	S	S	R	I	S	S	S	S	S	R	S	R	S	R	S	R	S
Buteo buteo	*Escherichia coli* [MDR]	R	S	S	S	S	S	R	I	S	R	R	S	S	R	S	R	S	R	R	S	S
Regulus regulus	*Escherichia coli*	S	S	S	S	S	S	R	I	S	S	S	S	S	R	R	R	S	S	S	S	S
Streptopelia decaocto	*Escherichia coli*	S	S	S	S	S	S	S	S	S	S	S	S	S	S	S	S	S	S	S	S	S
Streptopelia decaocto	*Escherichia coli*	S	S	S	S	S	S	S	S	S	S	S	S	S	S	S	S	S	S	S	S	S
Falco tinnunculus	*Escherichia coli*	S	S	S	S	S	S	R	I	S	S	S	S	S	R	R	R	S	S	S	S	S
Strix aluco	*Escherichia coli*	S	S	S	S	S	S	S	S	S	S	S	S	S	S	S	S	S	S	S	S	S
Scolopax rusticola	*Escherichia coli*	S	S	S	S	S	S	S	S	S	S	S	S	S	S	S	S	S	S	S	S	S
Gallinula chloropus	*Escherichia coli*	S	S	S	S	S	S	R	I	S	S	I	S	S	R	S	R	S	S	S	S	S
Sturnus vulgaris	*Escherichia coli*	S	S	S	S	S	S	S	S	S	S	S	S	S	S	S	S	S	S	S	I	S
Picus viridis	*Escherichia coli*	S	S	S	S	S	S	R	I	S	S	I	S	S	R	R	R	S	S	S	S	S

Sylvia atricapilla	*Escherichia coli*	S	S	S	S	S	S	S	S	S	S	S	S	S	S	S	S	S	S	S	S	S
Ardea cinerea	*Escherichia coli*	S	S	S	S	S	S	S	S	S	S	S	S	S	S	S	S	S	S	S	S	S
Otus scops	*Escherichia coli*	S	S	S	S	S	S	S	S	S	S	S	S	S	S	S	S	S	S	S	S	S
Bubulcus ibis	*Escherichia coli*	S	S	S	S	S	S	S	S	S	S	S	S	S	S	S	S	S	S	S	S	S
Athene noctua	*Escherichia coli*	S	S	S	S	S	S	S	S	S	S	S	S	S	S	S	S	S	S	S	S	S
Athene noctua	*Escherichia coli*	S	S	S	S	S	S	S	S	S	S	S	S	S	S	S	R	S	S	S	S	S
Corvus cornix	*Escherichia coli*	S	S	S	S	S	S	S	S	S	S	S	S	S	S	S	S	S	S	S	S	R
Corvus cornix	*Escherichia coli* ^{MDR}	R	S	S	S	S	S	R	R	S	R	R	S	S	R	S	R	S	R	R	S	S
Larus michahellis	*Escherichia coli* ^{MDR}	R	S	S	S	S	S	R	R	S	I	R	S	S	S	S	R	S	S	S	I	S
Larus michahellis	*Escherichia coli*	S	S	S	S	S	S	S	S	S	S	S	S	S	S	S	S	S	S	S	S	S
Fringilla coelebs	*Escherichia coli*	S	S	S	S	S	S	R	I	S	S	S	S	S	R	S	R	S	S	S	S	S
Upupa epops	*Escherichia coli*	S	S	S	S	S	S	S	S	S	S	S	S	S	S	S	S	S	S	S	S	S
Anas platyrhynchos	*Escherichia coli*	S	S	S	S	S	S	S	S	S	S	S	S	S	S	S	S	S	S	S	S	S
Falco tinnunculus	*Escherichia coli*	S	S	S	S	S	S	S	S	S	S	S	S	S	S	S	S	S	S	S	S	S
Falco tinnunculus	*Escherichia coli*	S	S	S	S	S	S	S	S	S	S	S	S	S	R	S	R	S	S	S	S	S
Turdus merula	*Escherichia coli*	S	S	S	S	S	S	S	S	S	S	S	S	S	S	S	S	S	S	S	S	S
Turdus merula	*Escherichia coli*	S	S	S	S	S	S	S	S	S	S	S	S	S	S	S	S	S	S	S	S	S
Turdus merula	*Escherichia coli*	S	S	S	S	S	S	S	S	S	S	S	S	S	S	S	S	S	S	S	S	S
Columba palumbus	*Escherichia coli*	S	S	S	S	S	S	S	S	S	S	S	S	S	R	R	R	S	R	S	S	S
Apus apus	*Escherichia coli*	S	S	S	S	S	S	R	I	S	S	S	S	S	R	S	R	S	S	S	S	S
Turdus merula	*Escherichia coli*	S	S	S	S	S	S	R	I	S	S	S	S	S	S	S	S	S	S	S	S	S
Apus apus	*Escherichia coli*	S	S	S	S	S	S	S	S	S	S	S	S	S	S	S	S	S	S	S	S	S
Phylloscopus collybita	*Escherichia coli* ^{MDR}	S	S	S	S	S	S	R	I	S	S	S	S	S	R	S	R	S	S	R	S	S
Turdus merula	*Escherichia coli*	S	S	S	S	S	S	S	S	S	S	S	S	S	S	S	S	S	S	S	S	S
Columba palumbus	*Escherichia coli*	S	S	S	S	S	S	S	S	S	S	S	S	S	S	S	S	S	S	S	S	S
Phoenicurus phoenicurus	*Escherichia coli* ^{MDR}	S	S	S	S	S	S	R	I	S	S	S	S	S	R	S	S	S	R	S	S	S
Serinus serinus	*Escherichia coli*	S	S	S	S	S	S	S	S	S	S	S	S	S	R	S	R	S	S	S	S	S
Pica pica	*Escherichia coli*	S	S	S	S	S	S	S	S	S	S	S	S	S	S	S	S	S	S	S	S	S
Streptopelia decaocto	*Escherichia coli*	S	S	S	S	S	S	S	S	S	S	S	S	S	S	S	S	S	S	S	S	S
Pica pica	*Escherichia coli*	S	S	S	S	S	S	S	S	S	S	S	S	S	S	S	S	S	S	S	S	S
Ficedula hypoleuca	*Escherichia coli*	S	S	S	S	S	S	S	S	S	S	S	S	S	S	S	S	S	S	S	S	S
Apus apus	*Escherichia coli*	S	S	S	S	S	S	S	S	S	S	S	S	S	S	S	S	S	S	S	S	S
Turdus merula	*Escherichia coli*	S	S	S	S	S	S	S	S	S	S	S	S	S	S	S	S	S	S	S	S	S
Garrulus glandarius	*Escherichia coli* ^{MDR}	S	S	S	S	S	S	R	R	S	S	I	S	S	R	S	R	S	R	S	S	S
Hirundo rustica	*Escherichia coli*	S	S	S	S	S	S	S	S	S	S	S	S	S	S	S	S	S	S	S	S	S
Tachymarptis melba	*Escherichia coli*	S	S	S	S	S	S	S	S	S	S	S	S	S	R	S	R	S	R	S	S	S
Apus apus	*Escherichia coli* ^{MDR}	S	S	S	S	S	S	R	I	S	S	S	S	S	R	S	R	S	S	R	S	R
Turdus merula	*Escherichia coli*	S	S	S	S	S	S	S	S	S	S	S	S	S	R	S	R	S	S	S	S	S
Columba palumbus	*Escherichia coli* ^{MDR}	S	S	S	S	S	S	R	I	S	S	S	S	S	R	S	R	S	R	S	S	S
Athene noctua	*Escherichia coli*	S	S	S	S	S	S	R	I	S	I	I	S	S	R	S	R	S	S	S	S	S

Serinus serinus	*Escherichia coli*	S	S	S	S	S	S	S	S	S	S	S	S	S	S	S	S	S	S	S	S	S
Turdus merula	*Escherichia coli* ^{MDR}	S	S	S	S	S	S	R	R	S	S	S	S	S	R	S	R	S	S	S	S	R
Streptopelia decaocto	*Escherichia coli*	S	S	S	S	S	S	R	I	S	S	S	S	S	S	S	S	S	S	S	S	S
Otus scops	*Escherichia coli*	S	S	S	S	S	S	S	S	S	S	S	S	S	S	S	S	S	S	S	S	S
Turdus merula	*Escherichia coli*	S	S	S	S	S	S	R	R	S	S	S	S	S	S	S	S	S	S	S	S	S
Athene noctua	*Escherichia coli*	S	S	S	S	S	S	S	S	S	S	S	S	S	R	S	R	S	S	S	S	S
Athene noctua	*Escherichia coli*	S	S	S	S	S	S	S	S	S	S	S	S	S	R	S	R	S	S	S	S	S
Falco tinnunculus	*Escherichia marmotae*	S	S	S	S	S	S	S	S	S	S	S	S	S	S	S	S	S	S	S	S	S
Sturnus vulgaris	*Escherichia marmotae*	S	S	S	S	S	S	S	S	S	S	S	S	S	S	S	S	S	S	S	S	S
Streptopelia decaocto	*Enterobacter hormaechei* ^{AmpC}	S	S	S	S	S	R^{IR}	S	S	S	S	S	S	S	R^{IR}	R^{IR}	S	S	S	S	S	S
Pica pica	*Enterobacter hormaechei* ^{AmpC}	S	S	S	S	S	R^{IR}	S	S	S	S	S	S	S	R^{IR}	R^{IR}	S	S	S	S	S	S
Fringilla coelebs	*Enterobacter hormaechei* ^{AmpC}	S	S	S	S	S	R^{IR}	S	S	S	S	S	S	S	R^{IR}	R^{IR}	S	S	S	S	S	S
Egretta garzetta	*Enterobacter hormaechei* ^{AmpC}	S	S	S	S	S	R^{IR}	S	S	S	S	S	S	S	R^{IR}	R^{IR}	S	S	S	S	S	S
Otus scops	*Enterobacter hormaechei* ^{AmpC}	S	S	S	S	S	R^{IR}	S	S	S	S	S	S	S	R^{IR}	R^{IR}	S	S	S	S	S	S
Serinus serinus	*Enterobacter roggenkampii* $^{AmpC; MDR}$	S	S	S	S	S	R^{IR}	R	I	S	S	S	S	S	R^{IR}	R^{IR}	S	S	R	R	S	S
Scolopax rusticola	*Hafnia alvei*	S	S	S	S	S	S^{IR}	S	S	S	S	S	S	S	R^{IR}	R^{IR}	S	S	S	S	S	R^{IR}
Picus viridis	*Hafnia alvei*	S	S	S	S	S	S^{IR}	S	S	S	S	S	S	S	R^{IR}	R^{IR}	S	S	S	S	I	R^{IR}
Athene noctua	*Hafnia alvei*	S	S	S	S	S	S^{IR}	S	S	S	S	S	S	S	S^{IR}	R^{IR}	S	S	S	S	I	R^{IR}
Hirundo rustica	*Hafnia alvei*	S	S	S	S	S	S^{IR}	S	S	S	S	S	S	S	S^{IR}	R^{IR}	S	S	S	S	S	R^{IR}
Pica pica	*Klebsiella oxytoca*	S	S	S	S	S	S	S	S	S^{IA}	S	S	S	S	R^{IR}	S	S	S	S	S	S	S
Turdus merula	*Klebsiella oxytoca*	S	S	S	S	S	S	S	S	S^{IA}	S	S	S	S	R^{IR}	S	S	S	S	S	S	S
Otus scops	*Klebsiella oxytoca*	S	S	S	S	S	S	S	S	S^{IA}	S	S	S	S	R^{IR}	S	S	S	S	S	S	S
Tachymarptis melba	*Klebsiella pneumoniae* $^{ESBL; MDR}$	S	R	R	R	S	S	R	I	R^{IA}	I	I	S	S	R^{IR}	S	R	S	R	S	R	R
Gallinula chloropus	*Klebsiella pneumoniae*	S	S	S	S	S	S	S	S	S^{IA}	S	S	S	S	R^{IR}	S	S	S	S	S	S	S
Turdus merula	*Klebsiella pneumoniae*	S	S	S	S	S	S	S	S	S^{IA}	S	S	S	S	R^{IR}	S	S	S	S	S	S	S
Turdus merula	*Klebsiella pneumoniae* ^{MDR}	S	S	S	S	S	S	R	R	R^{IA}	I	R	S	S	R^{IR}	R	R	R	S	S	S	S
Phoenicurus phoenicurus	*Klebsiella pneumoniae* $^{ESBL; MDR}$	S	R	R	R	S	S	S	S	S^{IA}	R	R	S	S	R^{IR}	S	R	R	S	S	R	S
Ficedula hypoleuca	*Klebsiella pneumoniae* $^{ESBL; MDR}$	R	R	I	R	S	S	R	R	S^{IA}	R	R	S	S	R^{IR}	R	R	S	R	S	R	S
Apus apus	*Klebsiella pneumoniae*	S	S	S	S	S	S	S	S	S^{IA}	S	S	S	S	R^{IR}	S	S	S	S	S	S	S
Tachymarptis melba	*Klebsiella pneumoniae* $^{ESBL; MDR}$	S	R	R	R	S	S	R	I	S^{IA}	I	R	S	S	R^{IR}	S	R	S	R	S	R	S
Tachymarptis melba	*Klebsiella pneumoniae*	S	S	S	S	S	S	S	S	S^{IA}	S	S	S	S	R^{IR}	S	S	S	S	S	S	R
Streptopelia decaocto	*Klebsiella pneumoniae*	S	S	S	S	S	S	S	S	S^{IA}	S	S	S	S	R^{IR}	S	S	S	S	S	S	S
Turdus merula	*Klebsiella aerogenes* ^{AmpC}	S	S	S	S	S	R^{IR}	S	S	S^{IA}	S	S	S	S	R^{IR}	R^{IR}	S	S	S	S	S	S
Streptopelia decaocto	*Leclercia adecarboxylata*	S	S	S	S	S	S	S	S	S	S	S	S	S	S	S	S	S	S	S	S	S
Fringilla coelebs	*Lelliottia amnigena* $^{AmpC; MDR}$	R	R	R	R	R	R	R	R	S	S	S	S	S	S	S	S	S	S	S	R	S
Pica pica	*Morganella morganii* ^{AmpC}	S	S	S	S	S	S	R^{IR}	R^{IR}	R^{IA}	S	S	S	I	R^{IR}	R^{IR}	S	S	S	S	S	R^{IR}
Upupa epops	*Morganella morganii* ^{AmpC}	S	S	S	S	S	S	R^{IR}	R^{IR}	R^{IA}	S	S	S	I	R^{IR}	R^{IR}	S	S	S	R	S	R^{IR}
Turdus merula	*Morganella morganii* ^{AmpC}	S	S	S	S	S	S	R^{IR}	R^{IR}	R^{IA}	S	S	S	I	R^{IR}	R^{IR}	S	S	S	S	S	R^{IR}
Turdus merula	*Proteus hauseri*	S	S	S	S	S	S	S	S	S^{IA}	S	S	S	I	R	S	S	S	S	S	S	R^{IR}
Hirundo rustica	*Proteus mirabilis*	S	S	S	S	S	S	R^{IR}	R^{IR}	S^{IA}	S	S	S	S	S	S	S	S	S	S	S	R^{IR}

Gallinula chloropus	*Proteus mirabilis*	S	S	S	S	S	S	R^{IR}	R^{IR}	S^{IA}	S	S	S	I	S	S	S	S	S	S	S	R^{IR}
Apus apus	*Proteus mirabilis*	S	S	S	S	S	S	R^{IR}	R^{IR}	S^{IA}	S	S	S	I	S	S	S	S	S	S	S	R^{IR}
Apus apus	*Proteus mirabilis*	S	S	S	S	S	S	R^{IR}	R^{IR}	S^{IA}	S	S	S	I	S	S	S	S	S	S	S	R^{IR}
Parus major	*Proteus mirabilis*	S	S	S	S	S	S	R^{IR}	R^{IR}	S^{IA}	S	S	S	I	S	S	S	S	S	S	S	R^{IR}
Ardea cinerea	*Proteus mirabilis*	S	S	S	S	S	S	R^{IR}	R^{IR}	S^{IA}	S	I	S	I	R	S	S	S	S	R	S	R^{IR}
Otus scops	*Proteus mirabilis*	S	S	S	S	S	S	R^{IR}	R^{IR}	S^{IA}	S	S	S	I	S	S	S	S	S	S	S	R^{IR}
Bubulcus ibis	*Proteus mirabilis*	S	S	S	S	S	S	R^{IR}	R^{IR}	S^{IA}	S	S	S	I	S	S	S	S	S	S	S	R^{IR}
Athene noctua	*Proteus mirabilis*	S	S	S	S	S	S	R^{IR}	R^{IR}	S^{IA}	S	S	S	I	S	S	S	S	S	S	S	R^{IR}
Athene noctua	*Proteus mirabilis*	S	S	S	S	S	S	R^{IR}	R^{IR}	S^{IA}	S	S	S	I	S	S	S	S	S	S	S	R^{IR}
Corvus cornix	*Proteus mirabilis*	S	S	S	S	S	S	R^{IR}	R^{IR}	S^{IA}	S	S	S	I	S	S	S	S	S	S	S	R^{IR}
Corvus cornix	*Proteus mirabilis*	S	S	S	S	S	S	R^{IR}	R^{IR}	S^{IA}	S	S	S	I	S	S	S	S	S	S	S	R^{IR}
Corvus cornix	*Proteus mirabilis* ^{MDR}	S	S	S	S	S	S	R^{IR}	R^{IR}	S^{IA}	S	S	S	I	R	S	S	S	R	R	S	R^{IR}
Pica pica	*Proteus mirabilis*	S	S	S	S	S	S	R^{IR}	R^{IR}	R^{IA}	S	S	S	I	R	S	S	S	S	S	S	R^{IR}
Anas platyrhynchos	*Proteus mirabilis*	S	S	S	S	S	S	R^{IR}	R^{IR}	S^{IA}	S	S	S	I	S	S	S	S	S	R	S	R^{IR}
Falco tinnunculus	*Proteus mirabilis*	S	S	S	S	S	S	R^{IR}	R^{IR}	S^{IA}	S	S	S	I	S	S	S	S	S	S	S	R^{IR}
Turdus merula	*Proteus mirabilis*	S	S	S	S	S	S	R^{IR}	R^{IR}	R^{IA}	S	S	S	I	S	S	S	S	S	S	I	R^{IR}
Turdus merula	*Proteus mirabilis*	S	S	S	S	S	S	R^{IR}	R^{IR}	S^{IA}	S	S	S	I	S	S	S	S	S	S	S	R^{IR}
Turdus merula	*Proteus mirabilis*	S	S	S	S	S	S	R^{IR}	R^{IR}	S^{IA}	S	S	S	I	S	S	S	S	S	R	S	R^{IR}
Tachymarptis melba	*Proteus mirabilis* ^{MDR}	S	S	S	S	S	S	R^{IR}	R^{IR}	S^{IA}	I	R	S	I	R	S	S	S	R	R	S	R^{IR}
Apus apus	*Proteus mirabilis*	S	S	S	S	S	S	R^{IR}	R^{IR}	S^{IA}	S	S	S	I	S	S	S	S	S	S	S	R^{IR}
Columba palumbus	*Proteus mirabilis*	S	S	S	S	S	S	R^{IR}	R^{IR}	R^{IA}	S	S	S	I	S	S	S	S	S	S	S	R^{IR}
Apus apus	*Proteus mirabilis*	S	S	S	S	S	S	R^{IR}	R^{IR}	S^{IA}	S	S	S	I	S	S	S	S	S	S	S	R^{IR}
Turdus merula	*Proteus mirabilis* ^{MDR}	S	S	S	S	S	S	R^{IR}	R^{IR}	S^{IA}	S	I	S	I	R	S	S	S	R	R	S	R^{IR}
Apus apus	*Proteus mirabilis*	S	S	S	S	S	S	R^{IR}	R^{IR}	S^{IA}	S	S	S	I	S	S	S	S	S	S	S	R^{IR}
Corvus cornix	*Proteus mirabilis*	S	S	S	S	S	S	R^{IR}	R^{IR}	S^{IA}	S	S	S	I	S	S	S	S	S	S	S	R^{IR}
Phoenicurus phoenicurus	*Proteus mirabilis*	S	S	S	S	S	S	R^{IR}	R^{IR}	R^{IA}	S	S	S	I	S	S	S	S	S	S	S	R^{IR}
Streptopelia decaocto	*Proteus mirabilis*	S	S	S	S	S	S	R^{IR}	R^{IR}	S^{IA}	S	S	S	I	S	S	S	S	S	S	S	R^{IR}
Turdus merula	*Proteus mirabilis*	S	S	S	S	S	S	R^{IR}	R^{IR}	S^{IA}	S	S	S	I	S	S	S	S	S	S	S	R^{IR}
Athene noctua	*Proteus mirabilis*	S	S	S	S	S	S	R^{IR}	R^{IR}	S^{IA}	S	S	S	I	S	S	S	S	S	S	S	R^{IR}
Turdus merula	*Proteus mirabilis*	S	S	S	S	S	S	R^{IR}	R^{IR}	S^{IA}	S	S	S	I	S	S	S	S	S	S	S	R^{IR}
Picus viridis	*Providencia rettgeri*	S	S	S	S	S	S	R^{IR}	R^{IR}	S^{IA}	S	S	S	I	R^{IR}	R^{IR}	S	S	S	S	S	R^{IR}
Streptopelia decaocto	*Pseudescherichia vulneris*	S	S	S	S	S	S	I	S	S	S	S	S	S	R	S	R	S	S	S	S	S
Athene noctua	*Salmonella* spp.	S	S	S	S	S	S	S	S	S	S	S	S	S	S	S	S	S	S	S	S	S
Athene noctua	*Salmonella* spp.	S	S	S	S	S	S	S	S	S	S	S	S	S	S	S	S	S	S	S	S	S
Otus scops	*Salmonella* spp.	S	S	S	S	S	S	S	S	S	S	S	S	S	S	S	S	S	S	S	S	S
Ardea cinerea	*Salmonella* spp.	S	S	S	S	S	S	S	S	S	S	S	S	S	S	S	S	S	S	S	S	S
Athene noctua	*Salmonella* spp.	S	S	S	S	S	S	S	S	S	S	S	S	S	S	S	S	S	S	S	S	S
Athene noctua	*Salmonella* spp.	S	S	S	S	S	S	S	S	S	S	S	S	S	S	S	S	S	S	S	S	S
Corvus cornix	*Salmonella* spp.	S	S	S	S	S	S	S	S	S	S	S	S	S	S	S	S	S	S	S	S	S
Pica pica	*Salmonella* spp.	S	S	S	S	S	S	I	S	S	S	S	S	S	R	S	R	S	S	R	S	S

Pica pica	*Salmonella* spp.	S	S	S	S	S	S	S	S	S	S	S	S	S	S	S	S	S	S	S	S	S
Falco tinnunculus	*Salmonella* spp.	S	S	S	S	S	S	S	S	S	S	S	S	S	S	S	S	S	S	S	S	S
Pica pica	*Salmonella* spp.	S	S	S	S	S	S	S	S	S	S	S	S	S	S	S	S	S	S	S	S	S
Falco tinnunculus	*Serratia liquefaciens*	S	S	S	S	S	S	S	S	S	S	S	S	S	S	S	S	S	S	S	S	R^{IR}
Falco tinnunculus	*Serratia marcescens*	S	S	S	S	S	S^{IR}	S	S	S	S	S	S	S	R^{IR}	R^{IR}	S	S	S	S	S	R^{IR}

R = resistant; I = intermediate resistant; S = susceptible; ^{IR} = intrinsic resistance according to EUCAST and/or CLSI [3,10]; ^{IA} = inadequate activity of the agent for the organism [1,11,12]; ^{ESBL} = ESBL-producing strains; ^{AmpC} = AmpC beta-lactamases-producing strains; ^{MDR} = Multi-drug resistant strain. Intrinsic resistances were not considered in the definition of the MDR profile [13]. CN: gentamicin, 10 μg; CTX: cefotaxime, 5 μg; CAZ: ceftazidime, 10 μg; FEP: cefepime, 30 μg; CZA: ceftazidime/avibactam, 10/4 μg; FOX: cefoxitin, 30 μg; TE: tetracycline, 30 μg; DO: doxycycline, 30 μg; TGC: tigecycline, 15 μg; LEV: levofloxacin, 5 μg; CIP: ciprofloxacin, 5 μg; MEM: meropenem, 10 μg; IPM: imipenem, 10 μg; AMP: ampicillin, 10 μg; AMC: amoxicillin/clavulanic acid, 20/10 μg; PRL: piperacillin, 30 μg; TZP: piperacillin/tazobactam, 30/6 μg; SXT: sulfamethoxazole/trimethoprim, 1.25/23.75 μg; C: chloramphenicol, 30 μg; ATM: aztreonam, 30 μg; CS: colistin, 0.016 - 256 μg/mL.

Table S4. Antimicrobial resistance profile of *Pseudomonadales* isolates. For all isolated bacterial strains, the interpretation of inhibition zones ("S", "I", "R") is reported based on breakpoints defined by EUCAST and CLSI. In the absence of species-specific breakpoints, those defined for related species or for species within the same taxonomic order were applied. Where no breakpoints were available for the agent and the organism, the measured inhibition zone diameter (in mm) is reported.

**Wild bird species**	**Isolated bacteria strains**	**Antimicrobial susceptibility testing panel**																
		Aminoglycosides	Cephalosporins				Tetracyclines		Glycylcyclines	Fluoroquinolones		Carbapenems		Penicillis		Sulphonamides	Monobactam	Polymyxin
		CN	CTX	CAZ	FEP	CZA	TE	DO	TGC	LEV	CIP	MEM	IPM	PRL	TZP	SXT	ATM	CS
Streptopelia decaocto	*Pseudomonas aeruginosa* [AmpC]	S[E; IE]	12[IR]	S	S	S	17[IR]	11[IR]	11[IR]	S	S	S	S	S	S	13[IR]	S	S
Parus major	*Pseudomonas aeruginosa* [AmpC]	S[E; IE]	13[IR]	S	S	S	10[IR]	10[IR]	10[IR]	S	S	S	S	S	S	10[IR]	S	S
Turdus merula	*Pseudomonas aeruginosa* [AmpC]	S[E; IE]	13[IR]	S	S	S	13[IR]	10[IR]	10[IR]	S	S	S	S	S	S	0[IR]	S	S
Hirundo rustica	*Pseudomonas aeruginosa* [AmpC]	S[E; IE]	14[IR]	S	S	S	14[IR]	10[IR]	10[IR]	S	S	S	S	S	S	10[IR]	S	S
Serinus serinus	*Pseudomonas aeruginosa* [AmpC]	S[E; IE]	14[IR]	S	S	S	14[IR]	0[IR]	13[IR]	S	S	S	S	S	S	10[IR]	S	S
Pica pica	*Acinetobacter baumannii*	S	R[IR]	I	I	21	20[IR]	23[IR]	22[IE]	S	S	S	S	S[IE]	S[IE]	S	16[IR]	S
Asio otus	*Acinetobacter pittii* [ESBL]	S	R[IR]	I	I	16	20[IR]	22[IR]	20[IE]	S	S	S	S	R[IE]	I[IE]	S	16[IR]	S

R = resistant; I = intermediate resistant; S = susceptible; [E] = Interpretation was based on the EUCAST ECOFFs defined for CN in the case of *Pseudomonas aeruginosa*; [IR] = intrinsic resistance according to EUCAST and/or CLSI [3,10]; [IE] = Insufficient evidence that the organism or group is a suitable target for therapy with the agent [1,11]; [ESBL] = ESBL-producing strains; [AmpC] = AmpC beta-lactamases-producing strains. CN: gentamicin, 10 µg; CTX: cefotaxime, 30 µg; CAZ: ceftazidime, 30 µg; FEP: cefepime, 30 µg; CZA: ceftazidime/avibactam, 10/4 µg; TE: tetracycline, 30 µg; DO: doxycycline, 30 µg; TGC: tigecycline, 15 µg; LEV: levofloxacin, 5 µg; CIP: ciprofloxacin, 5 µg; MEM: meropenem, 10 µg; IPM: imipenem, 10 µg; PRL: piperacillin, 100 µg; TZP: piperacillin/tazobactam, 100/10 µg; SXT: sulfamethoxazole/trimethoprim, 1.25/23.75 µg; ATM: aztreonam, 30 µg; CS: colistin, 0.016 - 256 µg/mL.

Table S5. Antimicrobial resistance profile of *Burkholderiales* isolates. For all isolated bacterial strains, the interpretation of inhibition zones ("S", "I", "R") is reported based on breakpoints defined by EUCAST and CLSI. In the absence of species-specific breakpoints, those defined for related species or for species within the same taxonomic order were applied. Where no breakpoints were available for the agent and the organism, the measured inhibition zone diameter (in mm), or the MIC value (μg/mL) with specific reference to colistin, is reported.

**Wild bird species**	**Isolated bacteria strains**	**Antimicrobial susceptibility testing panel**																				
		Aminoglycosides	Cephalosporins				Cephamycins	Tetracyclines		Glycylcyclines	Fluoroquinolones		Carbapenems		Penicillis				Sulphonamides	Phenicols	Monobactam	Polymyxin
		CN	CTX	CAZ	FEP	CZA	FOX	TE	DO	TGC	LEV	CIP	MEM	IPM	AMP	AMC	PRL	TZP	SXT	C	ATM	CS
Streptopelia decaocto	*Achromobacter mucicolens*	I[IA]	20[IA]	22	25	23	0[IA]	S	S	30	24[IA*]	25[IA*]	S	S	17[IA]	27	30	S	S	25	0[IA]	S[IA*]
Gallinula chloropus	*Comamonas kerstersii*	20	10	17	17	17	26	29	30	23	29	30	30	30	21	31	16	21	22	27	0	2

R = resistant; I = intermediate resistant; S = susceptible; [IA] = Wild-type strains belonging to the *Achromobacter* genus appear to exhibit phenotypic resistance to these antimicrobials, as reported in the scientific literature [6,14]; [IA*] = Intrinsically insufficient activity, as defined by EUCAST (considering *Achromobacter xylosoxidans*) [7]. CN: gentamicin, 10 μg; CTX: cefotaxime, 5 μg; CAZ: ceftazidime, 10 μg; FEP: cefepime, 30 μg; CZA: ceftazidime/avibactam, 10/4 μg; FOX: cefoxitin, 30 μg; TE: tetracycline, 30 μg; DO: doxycycline, 30 μg; TGC: tigecycline, 15 μg; LEV: levofloxacin, 5 μg; CIP: ciprofloxacin, 5 μg; MEM: meropenem, 10 μg; IPM: imipenem, 10 μg; AMP: ampicillin, 10 μg; AMC: amoxicillin/clavulanic acid, 20/10 μg; PRL: piperacillin, 30 μg; TZP: piperacillin/tazobactam, 30/6 μg; SXT: sulfamethoxazole/trimethoprim, 1.25/23.75 μg; C: chloramphenicol, 30 μg; ATM: aztreonam, 30 μg; CS: colistin, 0.016 - 256 μg/mL.

Table S6. Antimicrobial susceptibility (Kirby–Bauer method), number and percentage (in brackets) of resistant strains by antimicrobial agent and bacterial species within *Enterobacterales* order. Isolates showing intermediate susceptibility were considered susceptible for interpretation purposes, and therefore their number is not included in the table. The total number of strains resistant to the tested antimicrobial agents was determined excluding those whose resistance is intrinsically determined.

**Antimicrobial classes**	**Molecules**	**Isolated bacteria species**																				**Total number of resistant strains within the bacterial order** (n=148)
		C. braakii (n=2)	*C. freundii* (n=2)	*E. coli* (n=66)	*E. marmotae* (n=2)	*E. hormaechei* (n=5)	*E. roggenkampii* (n=1)	*H. alvei* (n=4)	*K. oxytoca* (n=3)	*K. pneumoniae* (n=10)	*K. aerogenes* (n=1)	*L. adecarboxylata* (n=1)	*L. amnigena* (n=1)	*M. morganii* (n=3)	*P. hauseri* (n=1)	*P. mirabilis* (n=31)	*P. rettgeri* (n=1)	*P. vulneris* (n=1)	*Salmonella* spp. (n=11)	*S. liquefaciens* (n=1)	*S. marcescens* (n=1)	
Aminoglycosides	CN	0	1 (50)	4 (6)	0	0	0	0	0	1 (10)	0	0	1 (100)	0	0	0	0	0	0	0	0	**7 (4.7)**
Cephalosporins	CTX	0	0	0	0	0	0	0	0	4 (40)	0	0	1 (100)	0	0	0	0	0	0	0	0	**5 (3.4)**
	CAZ	0	0	0	0	0	0	0	0	3 (30)	0	0	1 (100)	0	0	0	0	0	0	0	0	**4 (2.7)**
	FEP	0	0	0	0	0	0	0	0	4 (40)	0	0	1 (100)	0	0	0	0	0	0	0	0	**5 (3.4)**
	CZA	0	0	0	0	0	0	0	0	0	0	0	1 (100)	0	0	0	0	0	0	0	0	**1 (0.7)**
Cephamycins	FOX	2^{IR} (100)	2^{IR} (100)	0	0	5^{IR} (100)	1^{IR} (100)	0^{IR}	0	0	1^{IR} (100)	0	1 (100)	0	0	0	0	0	0^{NCE}	0	0^{IR}	**1 (0.7)**
Tetracyclines	TE	0	1 (50)	22 (33)	0	0	1 (100)	0	0	4 (40)	0	0	1 (100)	3^{IR} (100)	0	31^{IR} (100)	1^{IR} (100)	0	0	0	0	**29 (19.6)**
	DO	0	0	6 (9)	0	0	0	0	0	2 (20)	0	0	1 (100)	3^{IR} (100)	0	31^{IR} (100)	1^{IR} (100)	0	0	0	0	**9 (6.1)**
Glycylcyclines	TGC	0	0	0	0	0	0	0	0^{IA}	2^{IA} (20)	0^{IA}	0	0	3^{IA} (100)	0^{IA}	4^{IA} (12.9)	0^{IA}	0	0	0	0	**9 (6.1)**
Fluoroquinolones	LEV	0	0	3 (4.5)	0	0	0	0	0	2 (20)	0	0	0	0	0	0	0	0	0	0	0	**5 (3.4)**

	CIP	0	0	5 (7.6)	0	0	0	0	0	4 (40)	0	0	0	0	0	1 (3.2)	0	0	0	0	0	**10 (6.7)**
Carbapenems	MEM	0	0	0	0	0	0	0	0	0	0	0	0	0	0	0	0	0	0	0	0	**0**
	IPM	0	0	0	0	0	0	0	0	0	0	0	0	0	0	0	0	0	0	0	0	**0**
Penicillis	AMP	2^{IR} (100)	2^{IR} (100)	27 (40.9)	0	5^{IR} (100)	1^{IR} (100)	2^{IR} (50)	3^{IR} (100)	10^{IR} (100)	1^{IR} (100)	0	0	3^{IR} (100)	1 (100)	5 (16.1)	1^{IR} (100)	1 (100)	1 (9.1)	0	1^{IR} (100)	**35 (23.6)**
	AMC	2^{IR} (100)	2^{IR} (100)	4 (6)	0	5^{IR} (100)	1^{IR} (100)	4^{IR} (100)	0	2 (20)	1^{IR} (100)	0	0	3^{IR} (100)	0	0	1^{IR} (100)	0	0	0	1^{IR} (100)	**6 (4)**
	PRL	0	2 (100)	28 (42)	0	0	0	0	0	5 (50)	0	0	0	0	0	0	0	1 (100)	1 (9.1)	0	0	**37 (25)**
	TZP	0	0	0	0	0	0	0	0	2 (20)	0	0	0	0	0	0	0	0	0	0	0	**2 (1.3)**
Sulphonamides	SXT	0	1 (50)	9 (13.6)	0	0	1 (100)	0	0	3 (30)	0	0	0	0	0	3 (9.7)	0	0	0	0	0	**17 (11.5)**
Phenicols	C	0	1 (50)	4 (6)	0	0	1 (100)	0	0	0	0	0	0	1 (33)	0	6 (19.3)	0	0	1 (9.1)	0	0	**14 (9.5)**
Monobactam	ATM	0	1 (50)	1 (1.5)	0	0	0	0	0	4 (40)	0	0	1 (100)	0	0	0	0	0	0	0	0	**7 (4.7)**
Polymyxin	CS	0	0	4 (6)	0	0	0	4^{IR} (100)	0	2 (20)	0	0	0	3^{IR} (100)	1^{IR} (100)	31^{IR} (100)	1^{IR} (100)	0	0	1^{IR} (100)	1^{IR} (100)	**6 (4)**

^{IR} = intrinsic resistance according to EUCAST and/or CLSI [3,10]; ^{IA} = inadequate activity of the agent for the organism [1,11,12]; ^{NCE} = not clinically effective in vivo, regardless of in vitro susceptibility test results [3]. CN: gentamicin, 10 μg; CTX: cefotaxime, 5 μg; CAZ: ceftazidime, 10 μg; FEP: cefepime, 30 μg; CZA: ceftazidime/avibactam, 10/4 μg; FOX: cefoxitin, 30 μg; TE: tetracycline, 30 μg; DO: doxycycline, 30 μg; TGC: tigecycline, 15 μg; LEV: levofloxacin, 5 μg; CIP: ciprofloxacin, 5 μg; MEM: meropenem, 10 μg; IPM: imipenem, 10 μg; AMP: ampicillin, 10 μg; AMC: amoxicillin/clavulanic acid, 20/10 μg; PRL: piperacillin, 30 μg; TZP: piperacillin/tazobactam, 30/6 μg; SXT: sulfamethoxazole/trimethoprim, 1.25/23.75 μg; C: chloramphenicol, 30 μg; ATM: aztreonam, 30 μg; CS: colistin, 0.016 - 256 μg/mL.

Table S7. Antimicrobial susceptibility (Kirby–Bauer method), number and percentage (in brackets) of resistant strains by antimicrobial agent and bacterial species within *Pseudomonadales* order. Isolates showing intermediate susceptibility were considered susceptible for interpretation purposes, and therefore their number is not included in the table.

Antimicrobial classes	Molecules	Isolated bacteria species			Total number of resistant strains within the bacterial order (n=7)
		P. aeruginosa (n=5)	*A. baumannii* (n=1)	*A. pittii* (n=1)	
Aminoglycosides	CN	0[E; IE]	0	0	**0**
Cephalosporins	CTX	ND[IR]	1 (100)	1 (100)	**2 (28.6)**
	CAZ	0	0	0	**0**
	FEP	0	0	0	**0**
	CZA	0	ND	ND	**0**
Tetracyclines	TE	ND[IR]	ND[IR]	ND[IR]	**-**
	DO	ND[IR]	ND[IR]	ND[IR]	**-**
Glycylcyclines	TGC	ND[IR]	ND[IE]	ND[IE]	**-**
Fluoroquinolones	LEV	0	0	0	**0**
	CIP	0	0	0	**0**
Carbapenems	MEM	0	0	0	**0**
	IPM	0	0	0	**0**
Penicillis	PRL	0	0[IE]	1[IE] (100)	**1 (14.3)**
	TZP	0	0[IE]	0[IE]	**0**
Sulphonamides	SXT	ND[IR]	0	0	**0**
Monobactam	ATM	0	ND[IR]	ND[IR]	**0**
Polymyxin	CS	0	0	0	**0**

[E] = Interpretation was based on the EUCAST ECOFFs defined for CN in the case of *Pseudomonas aeruginosa*; [IR] = intrinsic resistance according to EUCAST and/or CLSI [3,10]; [IE] = Insufficient evidence that the organism or group is a suitable target for therapy with the agent [1,11]; ND = Not Defined due to the absence of established breakpoints for the agent and the organism or any closely related bacterial species. CN: gentamicin, 10 µg; CTX: cefotaxime, 30 µg; CAZ: ceftazidime, 30 µg; FEP: cefepime, 30 µg; CZA: ceftazidime/avibactam, 10/4 µg; TE: tetracycline, 30 µg; DO: doxycycline, 30 µg; TGC: tigecycline, 15 µg; LEV: levofloxacin, 5 µg; CIP: ciprofloxacin, 5 µg; MEM: meropenem, 10 µg; IPM: imipenem, 10 µg; PRL: piperacillin, 100 µg; TZP: piperacillin/tazobactam, 100/10 µg; SXT: sulfamethoxazole/trimethoprim, 1.25/23.75 µg; ATM: aztreonam, 30 µg; CS: colistin, 0.016 - 256 µg/mL.

Table S8. Antimicrobial susceptibility (Kirby–Bauer method), number and percentage (in brackets) of resistant strains by antimicrobial agent and bacterial species within *Burkholderiales* order. Isolates showing intermediate susceptibility were considered susceptible for interpretation purposes, and therefore their number is not included in the table.

Antimicrobial classes	Molecules	Isolated bacteria species		Total number of resistant strains within the bacterial order (n=2)
		Achromobacter mucicolens (n=1)	*Comamonas kerstersii* (n=1)	
Aminoglycosides	CN	0[IA]	ND	-
Cephalosporins	CTX	ND[IA]	ND	-
	CAZ	ND	ND	-
	FEP	ND	ND	-
	CZA	ND	ND	-
Cephamycins	FOX	ND[IA]	ND	-
Tetracyclines	TE	0[IA]	ND	-
	DO	0	ND	-
Glycylcyclines	TGC	ND	ND	-
Fluoroquinolones	LEV	0[IA*]	ND	-
	CIP	0[IA*]	ND	-
Carbapenems	MEM	0	ND	-
	IPM	0[IA*]	ND	-
Penicillis	AMP	ND[IA]	ND	-
	AMC	ND	ND	-
	PRL	ND	ND	-
	TZP	0	ND	-
Sulphonamides	SXT	0	ND	-

Phenicols	C	ND	ND	-
Monobactam	ATM	ND[IA]	ND	-
Polymyxin	CS	ND[IA*]	ND	-

[IA] = Wild-type strains belonging to the *Achromobacter* genus appear to exhibit phenotypic resistance to these antimicrobials, as reported in the scientific literature [6,14]; [IA*] = Intrinsically insufficient activity, as defined by EUCAST (considering *Achromobacter xylosoxidans*) [7]; ND = Not Defined due to the absence of breakpoints for the agent and the tested organism or any closely related species. CN: gentamicin, 10 µg; CTX: cefotaxime, 5 µg; CAZ: ceftazidime, 10 µg; FEP: cefepime, 30 µg; CZA: ceftazidime/avibactam, 10/4 µg; FOX: cefoxitin, 30 µg; TE: tetracycline, 30 µg; DO: doxycycline, 30 µg; TGC: tigecycline, 15 µg; LEV: levofloxacin, 5 µg; CIP: ciprofloxacin, 5 µg; MEM: meropenem, 10 µg; IPM: imipenem, 10 µg; AMP: ampicillin, 10 µg; AMC: amoxicillin/clavulanic acid, 20/10 µg; PRL: piperacillin, 30 µg; TZP: piperacillin/tazobactam, 30/6 µg; SXT: sulfamethoxazole/trimethoprim, 1.25/23.75 µg; C: chloramphenicol, 30 µg; ATM: aztreonam, 30 µg; CS: colistin, 0.016 - 256 µg/mL.

Table S9. Binary logistic regression for major pathogen isolation. *Hosmer–Lemeshow test: p = 0.629.*

Variable	OR	p-value
Age	0.55	0.058
Feeding behavior	1.11	0.696
Spatial behavior	1.33	0.580
Habitat	0.45	0.100

Table S10. Binary logistic regression for *Salmonella* spp. isolation. *Hosmer–Lemeshow test: p = 0.984.*

Variable	OR	p-value
Age	2.05	0.241
Feeding behavior	0.14	**0.004***
Spatial behavior	2.10	0.540
Habitat	12.29	0.063

*Bold means a p-value <0.05, so a statistically significant value

Table S11. Binary logistic regression for piperacillin resistance in *E. coli*. *Hosmer–Lemeshow test: p = 0.952.*

Variable	OR	p-value
Age	1.91	0.092
Feeding behavior	1.35	0.337
Habitat	0.49	0.243
Spatial behavior	0.28	**0.048***

*Bold means a p-value <0.05, so a statistically significant value

References

1. EUCAST. Breakpoint tables for interpretation of MICs and zone diameters; Version 15.0. Available online: https://www.eucast.org/fileadmin/src/media/PDFs/EUCAST_files/Breakpoint_tables/v_15.0_Breakpoint_Tables.pdf (accessed on 14 April 2025).
2. EUCAST. Breakpoint tables for interpretation of MICs and zone diameters; Version 14.0. Available online: https://www.eucast.org/fileadmin/src/media/PDFs/EUCAST_files/Breakpoint_tables/v_14.0_Breakpoint_Tables.pdf (accessed on 14 April 2025).
3. CLSI. *Performance Standards for Antimicrobial Susceptibility Testing*, 35th ed.; Clinical and Laboratory Standards Institute: Wayne, PA, USA, 2025; Volume M100.
4. CLSI. *Performance Standards for Antimicrobial Susceptibility Testing*, 34th ed.; Clinical and Laboratory Standards Institute: Wayne, PA, USA, 2024; Volume M100.
5. Liofilchem. MIC Test Strip - Interpretative criteria and quality control. Available online: https://www.liofilchem.net/pdf/mic/tabella_interpretazione.pdf (accessed on 14 April 2025).
6. Almuzara, M.; Limansky, A.; Ballerini, V.; Galanternik, L.; Famiglietti, A.; Vay, C. In vitro susceptibility of *Achromobacter* spp. isolates: comparison of disk diffusion, Etest and agar dilution methods. *Int. J. Antimicrob. Agents* **2010**, *35*, 68-71, doi:https://doi.org/10.1016/j.ijantimicag.2009.08.015.
7. EUCAST. *Achromobacter xylosoxidans* - Proposed clinical MIC breakpoints. Available online: https://www.eucast.org/fileadmin/src/media/PDFs/EUCAST_files/Consultation/2020/A._xylosoxidans_EUCAST_Breakpoint_consultation_201005.pdf (accessed on 30 April 2025).
8. Adeolu, M.; Alnajar, S.; Naushad, S.; S. Gupta, R. Genome-based phylogeny and taxonomy of the '*Enterobacteriales*': proposal for *Enterobacterales* ord. nov. divided into the families *Enterobacteriaceae*, *Erwiniaceae* fam. nov., *Pectobacteriaceae* fam. nov., *Yersiniaceae* fam. nov., *Hafniaceae* fam. nov., *Morganellaceae* fam. nov., and *Budviciaceae* fam. nov. *International Journal of Systematic and Evolutionary Microbiology* **2016**, *66*, 5575-5599, doi:https://doi.org/10.1099/ijsem.0.001485.
9. Janda, J.M.; Abbott, S.L. The changing face of the family *Enterobacteriaceae* (order: "*Enterobacterales*"): new members, taxonomic issues, geographic expansion, and new diseases and disease syndromes. *Clin. Microbiol. Rev.* **2021**, *34*, e00174-00120, doi:https://doi.org/ 10.1128/cmr.00174-20.
10. EUCAST. Expected resistant phenotypes; Version 1.2. Available online: https://www.eucast.org/fileadmin/src/media/PDFs/EUCAST_files/Expert_Rules/2023/Expected_Resistant_Phenotypes_v1.2_20230113.pdf (accessed on 17 April 2025).
11. Brown, D.F.J.; Canton, R.; MacGowan, A.P.; Mouton, J.W.; Rodloff, A.; Goldstein, F.; Odenholt, I.; Steinbakk, M.; Varaldo, P.; Hryniewicz, W.; et al. EUCAST Technical Note on tigecycline. *Clin. Microbiol. Infect.* **2006**, *12*, 1147-1149, doi:https://doi.org/10.1111/j.1469-0691.2006.01578.x.
12. EUCAST. Tigecycline: Rationale for EUCAST Clinical Breakpoints; Version 3.0. Available online: https://www.eucast.org/fileadmin/src/media/PDFs/EUCAST_files/Rationale_documents/Tigecycline_Rationale_Document_v3.0_20230404.pdf (accessed on 25 Aprile 2025).
13. Magiorakos, A.P.; Srinivasan, A.; Carey, R.B.; Carmeli, Y.; Falagas, M.E.; Giske, C.G.; Harbarth, S.; Hindler, J.F.; Kahlmeter, G.; Olsson-Liljequist, B.; et al. Multidrug-resistant, extensively drug-resistant and pandrug-resistant bacteria: an international expert proposal for interim standard definitions for acquired resistance. *Clin. Microbiol. Infect.* **2012**, *18*, 268-281, doi:https://doi.org/10.1111/j.1469-0691.2011.03570.x.
14. Isler, B.; Kidd, T.J.; Stewart, A.G.; Harris, P.; Paterson, D.L. *Achromobacter* infections and treatment options. *Antimicrob. Agents Chemother.* **2020**, *64*, e01025-01020, doi:https://doi.org/10.1128/aac.01025-20.
